# *Drosophila* phenylketonuria modeling helps reveal the disease etiology and the modulation role of iron

**DOI:** 10.1016/j.gendis.2025.101790

**Published:** 2025-08-07

**Authors:** Fan Du, Bing Zhou

**Affiliations:** a Key Laboratory of Quantitative Synthetic Biology, Shenzhen Institute of Synthetic Biology, Shenzhen Institutes of Advanced Technology, Chinese Academy of Sciences, Shenzhen, Guangdong 518055, China; b School of Life Sciences, Tsinghua University, Beijing 100084, China; c Faculty of Synthetic Biology, Shenzhen University of Advanced Technology, Shenzhen, Guangdong 518055, China

Phenylketonuria (PKU), one of the most common genetic metabolic disorders, is due to defective activity of phenylalanine hydroxylase (PAH), a member of the aromatic amino acid hydroxylases. In PKU patients, phenylalanine (Phe) levels in the blood and brain increase with clinical manifestations of severe intellectual disability and some other abnormalities. The exact mechanism and impacting factors of PKU are not completely elucidated. The therapy for PKU is mainly through a Phe-restricted diet to limit the uptake of Phe. In addition, tetrahydrobiopterin serves as an adjuvant pharmacologic therapy, primarily by its possible activity-boosting effect as a cofactor for PAH. Besides tetrahydrobiopterin, it is known that PAH additionally requires Fe^2+^ and O_2_ as cofactors (Fig. 1A). In this work, we generated a *D. melanogaster* PKU model to explore its regulatory mechanism. We found that the Phe-sensitivity of PKU *Drosophila* was strongly modulated by iron, and the eclosion defect could be almost completely rescued by tyrosine and L-3,4-dihydroxyphenylalanine (l-DOPA). The effect of iron on PKU was further confirmed in mouse PKU models.

**Figure 1:**
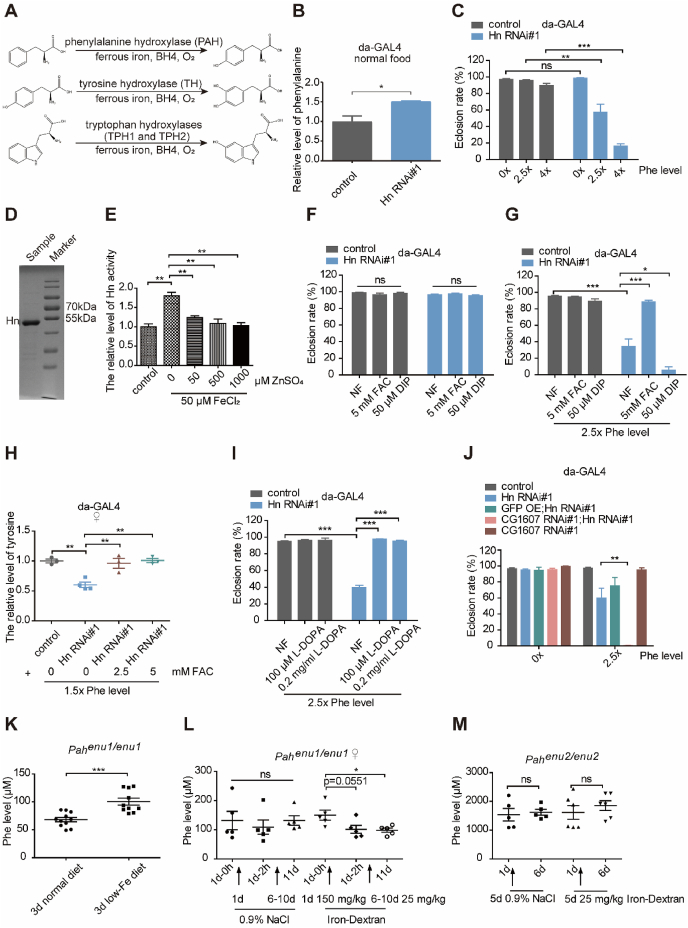
Regulation of iron on phenylalanine hydroxylase and phenylketonuria. **(A)** The reactions catalyzed by aromatic amino acid hydroxylases, including phenylalanine hydroxylase, tyrosine hydroxylase, and tryptophan hydroxylase. **(B)** Phe level was increased in *Hn*-knockdown *Drosophila*. *n* = 10 3rd-instar larvae per parallel and *n* = 3 parallel per experimental group. **(C)** Eclosion (the process from pupae to adult) rates of *da-GAL4* > control (TB072) and *da-GAL4* > *Hn RNAi#1* (THU2346) in foods with different Phe levels (0x, 2.5x, 4x). 1x Phe is 2.2 g/L. *n* = 60 larvae per vial and *n* ≥ 3 vials per experimental group. **(D)** The purified recombinant *Drosophila* phenylalanine hydroxylase (Hn) protein from *E. coli* was shown in a Coomassie blue staining of protein gel. **(E)** The effect that zinc antagonized iron in regulating Hn activity. **(F)** Eclosion rate of *da-GAL4* > control and *da-GAL4* > *Hn RNAi#1* in normal food (NF), 5 mM ferric ammonium citrate (FAC) food, and 50 μM 2,2'-bipyridyl (DIP) food. *n* = 60 larvae per vial and *n* ≥ 3 vials per experimental group. **(G)** 5 mM FAC rescued the eclosion defect of *da-GAL4* > *Hn RNAi#1* in 2.5x Phe food whereas 50 μM DIP exacerbated it. *n* = 60 larvae per vial and *n* ≥ 4 vials per experimental group. **(H)** FAC recovered the tyrosine level in the *Hn RNAi* flies' heads in 1.5x Phe food. *n* = 100 female flies' heads per parallel experiment, and *n* ≥ 3 parallels per experimental group. **(I)** The eclosion defect of ubiquitous *Hn* knockdown was rescued by l-DOPA. *n* = 60 larvae per vial and *n* = 4 vials per experimental group. **(J)***CG1607 RNAi* decreased the eclosion rate of *Hn RNAi* driven by *da-GAL4* in Phe food. *n* = 60 larvae per vial and *n* ≥ 3 vials per experimental group. **(K)** Phenylalanine levels of *Pah*^*enu1/enu1*^ mice increased after 3 days on the rodent diet with 25 ppm Fe in comparison to a 3-day normal diet. *n* = 10 or 11. **(L)** Changes of phenylalanine level in *Pah*^*enu1/enu1*^ mice after one iron-dextran intraperitoneal injection and five times of iron-dextran intraperitoneal injection. Mice were fed with the rodent diet, which contains 25 ppm Fe. *n* = 5. **(M)** Changes of phenylalanine level in *Pah*^*enu2/enu2*^ mice after five times of intraperitoneal iron-dextran injection. Mice were fed with the rodent diet. *n* = 5 or 6.

In the fruit fly, *Henna* (*Hn*) encodes a dual-function enzyme Phe/Trp (tryptophan) hydroxylase (Fig. 1A). Ubiquitous knockdown (RNAi) of *Hn* resulted in elevated bodily Phe level even when reared on normal food (Fig. 1B). These *Hn RNAi Drosophila* displayed increased sensitivity to extra Phe (Fig. 1C; Fig. S1A), leading to larval death (Fig. S1B), but exhibited no sensitivity to other large neutral amino acids (Fig. S1C), suggesting that *Hn* knockdown specifically sensitized *Drosophila* to Phe toxicity. *Hn* knockdown flies also exhibited motion defect (Fig. S1D, S1E). Because the *Drosophila* fat body is the functional homolog of the mammalian liver, where mammalian PAH is predominantly expressed, we also generated fat-specific *Hn* knockdown. Fat-driven *Hn*-knockdown *Drosophila* mimicked its universal knockdown (Fig. S2A), suggesting the fat body is where Phe to Tyr conversion primarily happens in the fly. Altogether, we successfully generated a PKU *Drosophila* model by knocking down *Hn*.

Regulation of PAH has long been a subject of study. Since tyrosine hydroxylase and PAH are both aromatic amino acid hydroxylases and require the same cofactors for hydroxylation reactions, given our prior research on metals (Fe/Zn) on tyrosine hydroxylase,^1^ we asked whether external Fe/Zn could influence Hn's PAH activity. To this end, recombinant *Drosophila* Hn protein was incubated with Fe^2+^ and Zn^2+^ (Fig. 1D). Fe^2+^ improved the Hn activity, whereas Zn^2+^ inhibited the Hn activity (Fig. S3A, S3B). The action of Zn^2+^ appears to be interfering with Fe^2+^ binding to Hn (Fig. S3C), likely by competing with it. Significantly, when Hn protein was co-incubated with both Fe^2+^ and Zn^2+^, Zn^2+^ could not effectively antagonize the Fe^2+^ under a high Fe^2+^ concentration (Fig. 1E). We then asked whether modulating Fe homeostasis could influence the survival of *Hn RNAi Drosophila*. When given a Phe diet, the eclosion defect resulting from Phe stress could very well be rescued by dietary iron supplementation (ferric ammonium citrate) and exacerbated by iron chelator 2,2'-bipyridyl (Fig. 1F, G; Fig. S4A–S4C). Virtually identical results were obtained with the fat body-specific *Hn* knockdown *Drosophila* (Fig. S2B, S2C). When adult flies were reared on the Phe food, both the wild-type and *Hn RNAi* adult flies exhibited reduced lifespans (Fig. S4D). Significantly, ferric ammonium citrate could partially rescue the longevity of both types of flies. Furthermore, iron addition made the lifespan difference between the wild-type and *Hn RNAi* flies indistinguishable. Thus, the critical defects, because of Hn loss, could be largely remedied by iron supplements. Direct ascertainment of Tyr levels indicated that under regular food rearing, *Hn* knockdown in the whole body indeed reduced the Tyr level and the PAH activity (Fig. S5A–S5D). Ferric ammonium citrate was able to significantly boost the Tyr level in *Hn* knockdown flies, in accordance with its rescue of the eclosion rate (Fig. S5E, S5F). A similar result was obtained when only heads were examined (Fig. 1H; Fig. S5G). Compared with the iron effect, the impact of zinc homeostasis on *Hn*-knockdown *Drosophila* was significantly more modest. The Phe food, zinc chelator TPEN, short for N,N,N',N'-tetrakis (2-pyridylmethyl)ethylenediamine, could partially rescue the eclosion defect of *Hn* knockdown *Drosophila* (Fig. S2D, S6A). However, ZnSO_4_ did not appreciably aggravate the eclosion defect. Genetic means to alter zinc homeostasis resulted in similar observations (Fig. S2E, S6B, S6C), suggesting that *in vivo*, zinc's effect is marginal.

One potential mechanism of impairment in PKU is that elevated blood Phe levels would competitively impair the import of other amino acids, such as Tyr and Trp, into the brain. The eclosion defect of *Hn RNAi Drosophila* could be almost entirely rescued by Tyr (Fig. S7A, S7B). In contrast, Trp had no rescue and, if anything, aggravated the defect. Other large neutral amino acids did not rescue the eclosion defect either (Fig. S7C). l-DOPA, the product of Tyr catalyzed by tyrosine hydroxylase, was also effective in improving *Hn* knockdown *Drosophila* (Fig. 1I; Fig. S7D), illustrating that Tyr and l-DOPA shortages are the key defective elements in the PKU *Drosophila*. In *Drosophila*, the two closest homologs of human LAT1/SLC7A5, the L-type amino acid transporter responsible for the uptake of large neutral amino acids, are *CG1607* and *CG12317*. Only *CG1607 RNAi* dramatically decreased the eclosion rate of *Hn RNAi Drosophila* in the Phe food (Fig. 1J). Notably, both tyrosine and l-DOPA could remarkably rescue the defect (Fig. S8A). We infer that CG1607 is the main tyrosine transporter to the brain.

Two often used mammalian PKU models, *Pah*^*enu1/enu1*^ and *Pah*^*enu2/enu2*^ mice, present a mild hyperphenylalaninemia and a severe PKU phenotype, respectively. We intraperitoneally injected the *Pah*^*enu1/enu1*^ mice with the iron chelator deferiprone. The blood Phe level dramatically increased shortly after deferiprone injection (Fig. S9A). On a more extended basis, intraperitoneal injection of deferiprone for three days also raised the Phe level (Fig. S9B). To investigate whether injection of iron-dextran could decrease the Phe level to improve PKU, we fed the animals with a 25 ppm Fe chow, a diet that is adequate in iron but not excessive. As a result, the Phe level in the blood of *Pah*^*enu1/enu1*^ mice under this diet was elevated, compared with *Pah*^*enu1/enu1*^ mice under the other normal diet with about 100 ppm Fe (Fig. 1K). Intraperitoneal injection of iron-dextran decreased the serum Phe level (Fig. 1L). This occurred after one injection of 150 mg/kg or a five-day injection of 25 mg/kg iron-dextran. Significantly, the injection of iron-dextran to *Pah*^*enu2/enu2*^ mice conferred no rescue on the Phe level (Fig. 1M). This result is an excellent control to show that the Phe reduction by iron is through the PAH activity, because *Pah*^*enu2/enu2*^ is possibly a null allele where the iron effect is unachievable. We also explored the possible impact of zinc on the blood Phe level; no significant changes were observed (Fig. S10A, S10B). Besides, dithizone, a zinc-chelating agent, could not change the blood Phe level of *Pah*^*enu1/enu1*^ mice (Fig. S10C).

These results demonstrated the validity of using a primitive organism, and in this case, the fruit fly, for PKU studies. However, subtle alterations, including mental abilities (*e.g.*, intelligence), are also possible but more difficult to ascertain in the fly. Previous studies show that zinc is a potent regulator of the activity of tyrosine hydroxylase, here, zinc supplement/chelation is not an efficient strategy for modifying PAH. This is likely due to the inefficient ability of zinc to compete against iron in binding PAH under physiological settings.

## CRediT authorship contribution statement

**Fan Du:** Visualization, Methodology, Data curation, Writing – original draft, Software, Formal analysis, Validation, Investigation. **Bing Zhou:** Writing – review & editing, Funding acquisition, Project administration, Supervision, Conceptualization.

## Ethics declaration

All experiments were approved by the Institutional Animal Care and Use Committee (IACUC) of Tsinghua University (Beijing, China) (Animal Protocol: 19-ZB1).

## Funding

This study was supported by the 10.13039/501100001809National Natural Science Foundation of China (No. 32371226) and the Shenzhen Medical Academy of Research and Translation (SMART) (Guangdong, China) (No. B2402001).

## Conflict of interests

No competing financial interests should be declared.
