# Supplementary material for: Drosophila phenylketonuria modeling helps reveal the disease etiology and the modulation role of iron
Source: Genes Dis. 2025 Aug 7;13(2):101790. doi: 10.1016/j.gendis.2025.101790 (PMC12664607; doi:10.1016/j.gendis.2025.101790)
Supplement: Multimedia component 1 [file mmc1.docx]

**Supplementary Materials**

**Materials and methods**

***Drosophila* strains and culture media**

*Drosophila* strains used in the study are listed as follows：

*da-GAL4* (Bloomington#8641) was from the Bloomington *Drosophila* Stock Center. *Cg-GAL4* was a present from Dr. Jose´Carlos Pastor-Pareja, Tsinghua University^1^. *Hn RNAi#1* (THU2346), *CG1607 RNAi#1* (TH04347.N) and TB072 were obtained from the Tsinghua Fly Center. *Hn RNAi#2* (V#35240) and *VDRC#w^1118^* were from the Vienna *Drosophila* Resource Center. Other transgenic fly strains used in this report have been previously described, including *dZnT1 RNAi*^2^, *dZip1 OE*^3^, *dZnT1 OE*^2^, *dZip1 RNAi*^4^, *dZip2 OE*^4^ and *dZip3-egfp OE*^5^.

All flies were maintained on standard cornmeal media at 25℃ with 60% humidity unless otherwise stated. Supplemented amino acids used were as follows: Phenylalanine (Phe), Tyrosine (Tyr), Tryptophan (Trp), Histidine (His), Valine (Val), Leucine (Leu) and Isoleucine (Ile). L-DOPA (3,4-Dihydroxyphenylalanine) was also used when necessary. 1x content in the diet equals 2.2g/L. When noted, the food was supplemented with metals and metal chelators, including ferric ammonium citrate (FAC; Sigma-Aldrich, Saint Louis, MO, USA), 2,2'-Bipyridyl (DIP; Aladdin, shanghai, China), N,N,N',N'-tetrakis (2-pyridylmethyl)ethylenediamine (TPEN; TCI (Shanghai) Development Co., Ltd., Shanghai, China) and Zinc sulfate heptahydrate (ZnSO_4_·7H_2_O; Sigma-Aldrich, Saint Louis, MO, USA) at concentrations as stated in each experiment.

**Eclosion, larvae survival, movement, and longevity assays**

For eclosion assays, different GAL4 lines were crossed with various transgenic lines for 2~3 days, as indicated in each experiment. Then the flies were allowed to lay eggs on juice-agar plates. Newly hatched first-instar larvae were transferred to normal food or food supplemented with different amino acids, metals or metal chelators, as indicated. The density of each vial was controlled to 60 larvae. The total number of emerged adult flies of each genotype was counted. Eclosion rate is ratio of the emerged adults to placed original 1^st^ instar larvae.

To assay larvae survival, 40 first-instar larvae newly hatched within 4 h were collected and placed in different foods, as indicated. We recorded the stage when the eggs hatch into the first-instar larva as 0 h. Larvae were suspended with 20% sucrose solution at 24 h, 48 h, 72 h, 96 h, 120 h and 144 h. The number of alive larvae was counted and confirmed by stereo-microscope. At least 4 tubes were checked at each time point. The larvae survival rate is the ratio of survived larvae to the initial number at specific moments as mentioned above.

For movement assays, 20 flies were placed in a glass tube. After 20 min recovery from CO_2_ exposure, flies were gently tapped to the bottom of tube. The number of flies climbing over 7 cm was counted after 7 s of climbing. Each tube was tested for 3 times and at least in 6 parallels. Climbing ability is the ratio of the counted number to the total number.

For longevity assays, 60 first-instar larvae were placed into different foods per vial. After eclosion, the female and male flies were transferred to fresh media respectively. The flies were moved to fresh food every few days and the number of alive flies were counted. Five parallel groups tests were conducted for each genotype.

**Phe level of *Drosophila***

10 third-instar larvae were lysed in PBS buffer, which contains 1% Triton X-100, 0.1 mg/ml PTU and protease inhibitor. The extract was centrifuged at 13000 rpm for 10 min in 4℃ twice and the supernatant was collected. Protein concentration was measured by the BCA Protein Assay Kit (Beyotime, Shanghai, China). The supernatant was collected by filter paper and Phe levels were measured by tandem mass spectrometry (TMS).

**Determination of tyrosine level and Hn activity in *Drosophila***

The tyrosine level and Hn activity were determined as previously described with slight modifications^6,7^. For tyrosine determination in *Drosophila* larvae, 10 third-instar larvae were lysed in the 0.1 M potassium phosphate (pH7.0), which contained 1 mM dithiothreitol (DTT) and Protease Inhibitor. For adults, 10 adult flies and 100 heads of flies were used to examine the tyrosine level. The extract was centrifuged at 10000 rpm for 15 min in 4℃ and the supernatant was collected. The supernatant was centrifuged again for 10 min and transferred to a new tube. The protein concentration was measured by the Bradford Protein Assay Kit (Beyotime, Shanghai, China). One part of the supernatant (50 μl) was used to detect the starting tyrosine level: mix the supernatant with 50 μl ddH_2_O and 0.1 ml ice-cold 15% trichloroacetic acid (TCA) and then collect 150 μl supernatant after 12000 rpm centrifugation for 3 min, the supernatant then reacted with nitrosonapthol reagent (300 μl) at 55℃ for 30 min with light shaking. The nitrosonaphthol reagent included the following components: 2 parts of 3N HNO_3_, 2 parts of 2.5% sodium nitrite, 1.3 parts of 0.5 mg/ml (in 95% ethanol) nitrosonaphthol, and 1 part of 95 % ethanol. Tecan infinite 200 Pro was used to measure the fluorescence. Measurement was performed with excitation at 465 nm and emission at 565 nm. The tyrosine level was calculated by comparing the measured value with the standard curve of tyrosine. The other part of supernatant was used to measure PAH activity. The assay mixture (0.2 ml total volume, pH7.0) contained the following components: 0.4 μmol phenylalanine, 0.064 μmol 6, 7-dimethyl 5, 6, 7, 8-tetrahydropterin (DMPH_4_), 0.1 μmol dithiothreitol and 10 μmol potassium phosphate. DMPH_4_ was the last one to add in the reaction system. The reaction proceeded in open tubes at 25℃ for 25 min. Ice-cold 15% TCA was added to terminate the reaction and then the mixture was centrifuged. The supernatant reacted with nitrosonapthol reagent and measured as described before. The difference of tyrosine values between before and after reaction could be regarded as Hn activity.

**Enzyme activity assay of Hn protein purified from *E. coli***

A double-tagged *Drosophila* Hn protein expression plasmid containing 6XHis and the HRV3C tag (pET28a-Hn-HRV3C-His) at the C-terminal was constructed. The ensuing plasmid was transformed into *E. coli* Rosseta2 DE3. Protein expression was induced by 1mM Isopropyl β-D-1-thiogalactopyranoside (IPTG) at 30℃ about 6 hours. The Hn-HRV3C-His fusion protein was purified with Ni-NTA resin, and the tags were removed by Recombinant Human Rhinovirus (HRV 3C) Protease (Sangon Biotech, Shanghai, China) digestion. The tags subsequently were removed by Ni-NTA. EDTA was added to a final concentration of 1mM to remove divalent metal iron. After incubation at 4℃ for 30min, the purified protein was concentrated by Amino Ultra Centrifugal Filters (type 30000 NMWL). Later, the protein was diluted 10-flod with 0.1M potassium phosphate (pH7.0) and concentrated to its initial volume by Ultra filters. The procedure was repeated for five times to remove the EDTA. Hn protein was detected by SDS-PAGE and coomassie blue staining. The collected Hn protein was then assayed for its activity. Briefly, protein Hn was incubated with FeCl_2_ or ZnSO_4_ for 30 min at 4℃ according to experimental requirements. After incubation, the treated protein sample was used to react with Phe as previously described. The protein which was not incubated with iron or zinc acted as the control. Tecan infinite 200 Pro was used to detect the newly formed tyrosine, which is regarded as the outcome of the Hn activity.

**Metal ion competition assay of Hn**

The assay was performed as previously described with some modification^8^. Purified Hn protein (2.5 μM) was incubated with 15 μM FeSO_4_, 0-15 μM ZnSO_4_, and 0.5 mg/mL catalase in 50 mM K/MES buffer (pH 6.8) for 20 min at 4 °C. After incubation, free and bound metal ions were separated using Amicon Ultra filter devices (Merck Millipore). The filtrate was collected and iron was determined by ICP-MS.

**Serum Phe level of PKU mice**

The PKU mice strains used in our study were *Pah^enu1/enu1^* and *Pah^enu2/enu2^* from The Jackson Laboratory. Deferiprone (DFP) was intraperitoneally injected to mice to check the short-term or long-term effects of iron-deficiency on serum Phe. To detect the effect of iron supplement on *Pah^enu1/enu1^* and *Pah^enu2/enu2^*, mice were fed on the Rodent Diet with 25 ppm Fe for some time, and were then treated with Iron-Dextran by intraperitoneal injection. In order to check zinc’s influence, mice were fed on the Rodent Diet with 25 ppm Fe and treated with ZnSO_4_ by intraperitoneal injection or intragastric administration, and dithizone by intraperitoneal injection. Saline solution was used as the control group for all experiments. Blood sample was taken from the tail tip to filter paper after 1 hour fasting. Phe levels were measured by tandem mass spectrometry (TMS). All experiments were approved by the Institutional Animal Care and Use Committee (IACUC) of Tsinghua University (Beijing, China) (Animal Protocol: 19-ZB1).

**Statistical analysis**

All data were analyzed by Student’s t-test in GraphPad Prism. Statistical results were presented as means ± SEM. Asterisks indicate critical levels of significance (*P < 0.05, **P < 0.01, ***P < 0.001).

**References**

1. Ke H, Feng Z, Liu M, et al. Collagen secretion screening in Drosophila supports a common secretory machinery and multiple Rab requirements. *Journal of genetics and genomics.* 2018;45(6):299-313.

2. Wang X, Wu Y, Zhou B. Dietary zinc absorption is mediated by ZnT1 in Drosophila melanogaster. *Faseb j.* 2009;23(8):2650-2661.

3. Lang M, Wang L, Fan Q, et al. Genetic inhibition of solute-linked carrier 39 family transporter 1 ameliorates aβ pathology in a Drosophila model of Alzheimer's disease. *PLoS Genet.* 2012;8(4):e1002683.

4. Qin Q, Wang X, Zhou B. Functional studies of Drosophila zinc transporters reveal the mechanism for dietary zinc absorption and regulation. *BMC Biol.* 2013;11:101.

5. Dechen K, Richards CD, Lye JC, Hwang JE, Burke R. Compartmentalized zinc deficiency and toxicities caused by ZnT and Zip gene over expression result in specific phenotypes in Drosophila. *Int J Biochem Cell Biol.* 2015;60:23-33.

6. Geltosky JE, Mitchell HK. Developmental regulation of phenylalanine hydroxylase activity in Drosophila melanogaster. *Biochem Genet.* 1980;18(7-8):781-791.

7. McCormick B, Young SK, Woods MN. Specificity of the colorimetric assay of tyrosine with i-nitroso-2-naphthol. *Clin Chim Acta.* 1965;12(2):216-218.

8. Xiao G, Zhao M, Liu Z, Du F, Zhou B. Zinc antagonizes iron-regulation of tyrosine hydroxylase activity and dopamine production in Drosophila melanogaster. *BMC Biol.* 2021;19(1):236.

**Supplementary Figures**

**
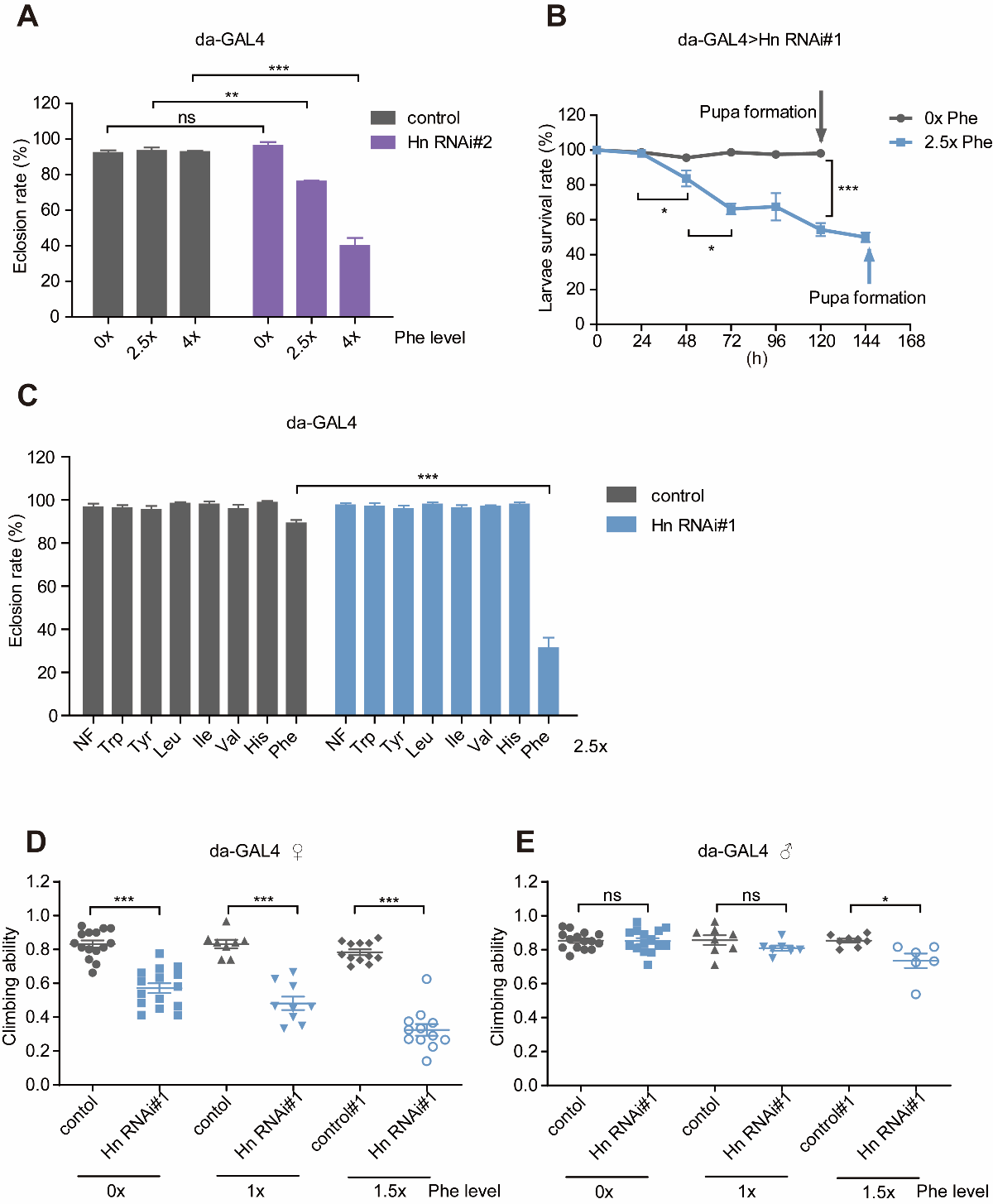
**

**Fig.S1 Ubiquitous *Hn* knockdown confers *Drosophila* phenylalanine sensitivity. (A)** Eclosion rates of *da-GAL4* > control (V#*w^1118^*) and *da-GAL4* > *Hn RNAi#2* (V#35240) in 0x, 2.5x and 4x Phe foods. n = 60 larvae per vial, n ≥ 3 vials per experimental group. **(B)** Larval lifespan of *Hn* knockdown. 0 h demotes the time when the eggs hatched to the 1^st^-instar larvae. n = 40 larvae per vial, n ≥ 4 vials per experimental group. **(C)** Eclosion rate of *da-GAL4* > control and *da-GAL4* > *Hn RNAi#1* in food with 2.5x different amino acids including Trp, Tyr, Leu, Ile, Val, His and Phe. n = 60 larvae per vial, n = 3 vials per experimental group. **(D)** *Hn*-konckdown flies displayed climbing defects in 0x, 1x and 1.5x Phe food. n=20 female flies per vial, n≥8 parallels per experimental group. **(E)** *Hn*-konckdown male flies show slightly climbing defects in 1.5x Phe food. n=20 male flies per vial, n≥6 parallels per experimental group.


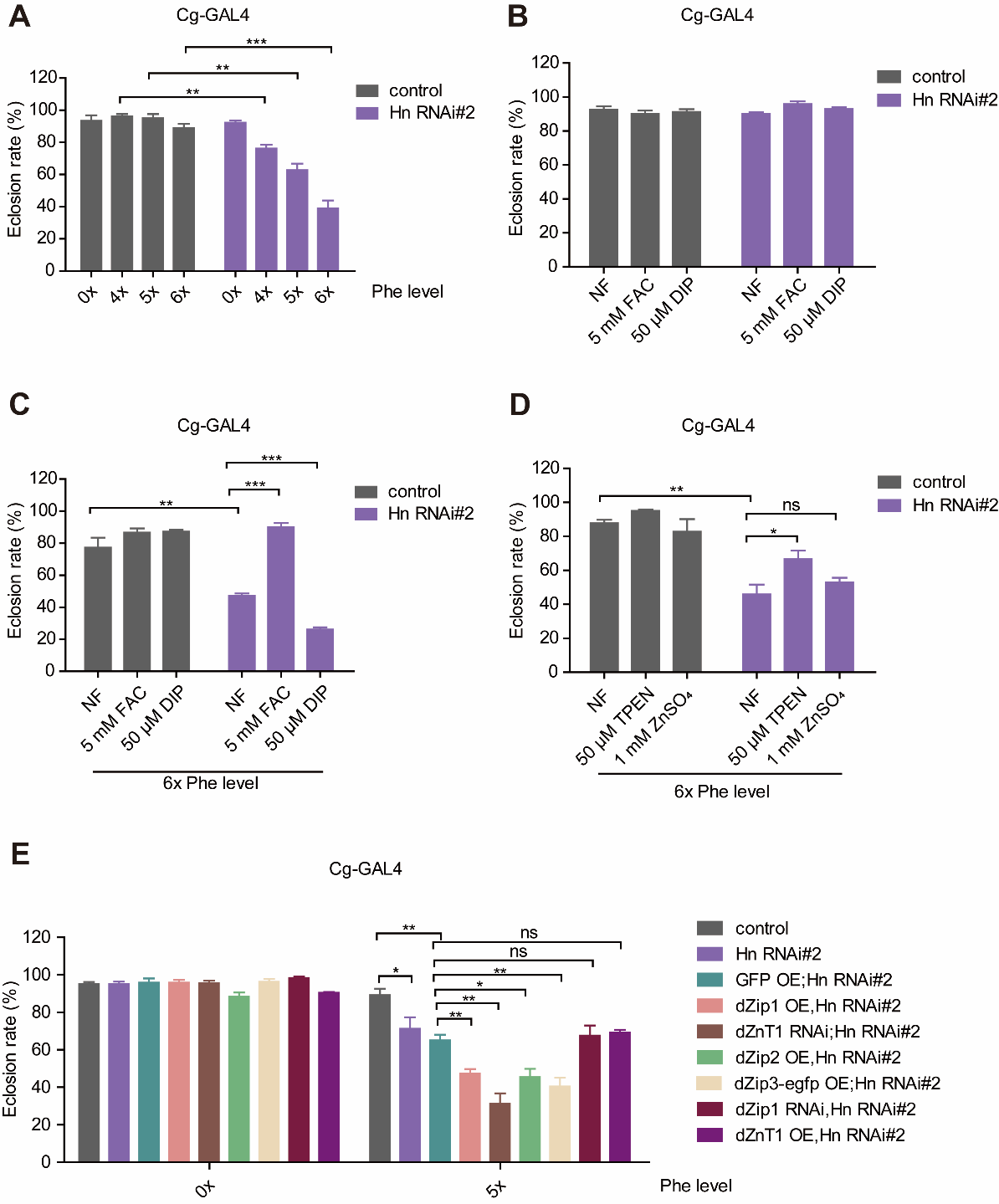


**Fig.S2 *Hn* knockdown in *Drosophila* fat body reproduces the phenotypes of the whole-body knockdown. (A)** Fat-specific Hn knockdown subjected *Drosophila* to Phe toxicity. Eclosion rates of *Cg-GAL4* > control (V#*w^1118^*) and *Cg-GAL4* > *Hn RNAi#2* (V#35240) in food with different Phe levels (0x, 4x, 5x, 6x) were analyzed. n = 60 larvae per vial, n = 3 vials per experimental group. **(B)** In normal food, dietary iron did not affect the eclosion of the fat-specific *Hn* knockdown *Drosophila*. *Cg-GAL4* > control and *Cg-GAL4* > *Hn RNAi#2 Drosophila* were tested*.* DIP was used the iron chelator. n = 60 larvae per vial, n = 4 vials per experimental group. **(C)** FAC supplement rescued, while iron chelation worsened, the eclosion defect of the fat-specific *Hn*-knockdown *Drosophila*. *Cg-GAL4* > control and *Cg-GAL4* > *Hn RNAi#2* in 6x Phe food was tested. n = 60 larvae per vial, n = 3 vials per experimental group. **(D)** Zinc modulation only mildly influenced the fat-specific *Hn*-knockdown *Drosophila*. 50 μM TPEN could slightly rescue the eclosion rate of *Cg-GAL4* > *Hn RNAi#2* in 6x Phe food*.* n = 60 larvae per vial, n ≥ 3 vials per experimental group. **(E)** The eclosion defect of the fat-specific *Hn RNAi* *Drosophila* could be moderately aggravated by zinc accumulation mediated by genetic measures. *Drosophila* was tested on 5x Phe food and the zinc homeostasis was interfered with *dZip1* OE, *dZnT1* RNAi, *dZip2* OE, *dZip3-egfp* OE, *dZip1 RNAi or dZnT1 OE*. Genotypes of the flies used were *Cg-GAL4* > control, *Cg-GAL4* > *Hn RNAi#2*, *Cg-GAL4* > *GFP OE; Hn RNAi#2*, *Cg-GAL4* > *dZip1 OE, Hn RNAi#2*, *Cg-GAL4* > *dZnT1 RNAi; Hn RNAi#2*, *Cg-GAL4* > *dZip2 OE, Hn RNAi#2*, *Cg-GAL4* > *dZip3-egfp OE; Hn RNAi#2*, *Cg-GAL4 > dZip1 RNAi, Hn RNAi#2*, *Cg-GAL4 > dZnT1 OE, Hn RNAi#2.* n = 60 larvae per vial, n = 4 vials per experimental group.


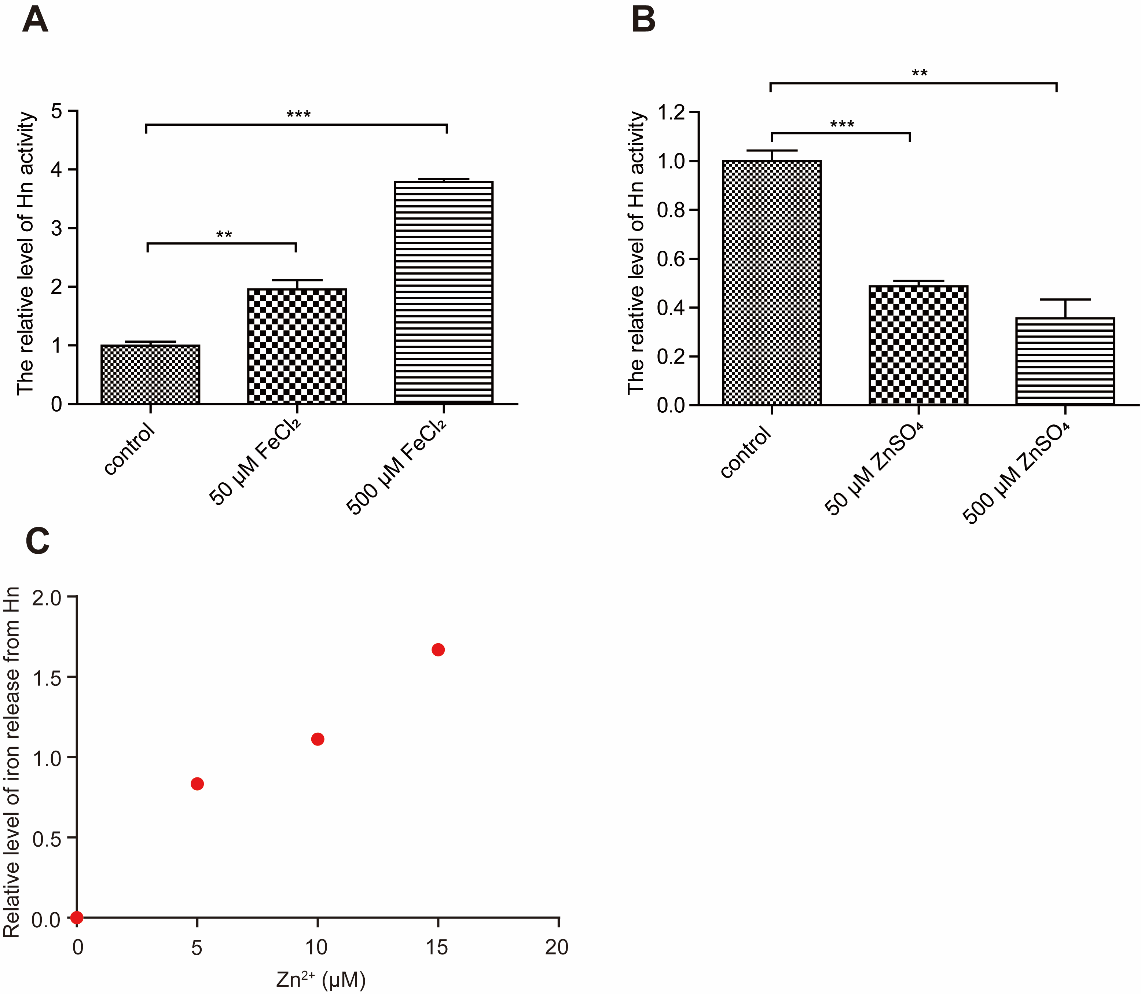


**Fig.S3 Hn activity is potently regulated by iron and to a lesser extent by zinc *in vitro*.** (**A)** The activity of recombinant Hn from *E. coli* was up-regulated by Fe^2+^. **(B)** The activity of recombinant Hn from *E. coli* was down-regulated by Zn^2+^. **(C)** Iron ion release from Hn by zinc ion. Hn was incubated with 15 μM ferrous iron and different concentrations of Zn^2+^. Free and bound metal ions were separated by ultrafiltration, and the iron level in filtrate was determined by ICP-MS. Free iron increased with increased zinc concentrations in the buffer.


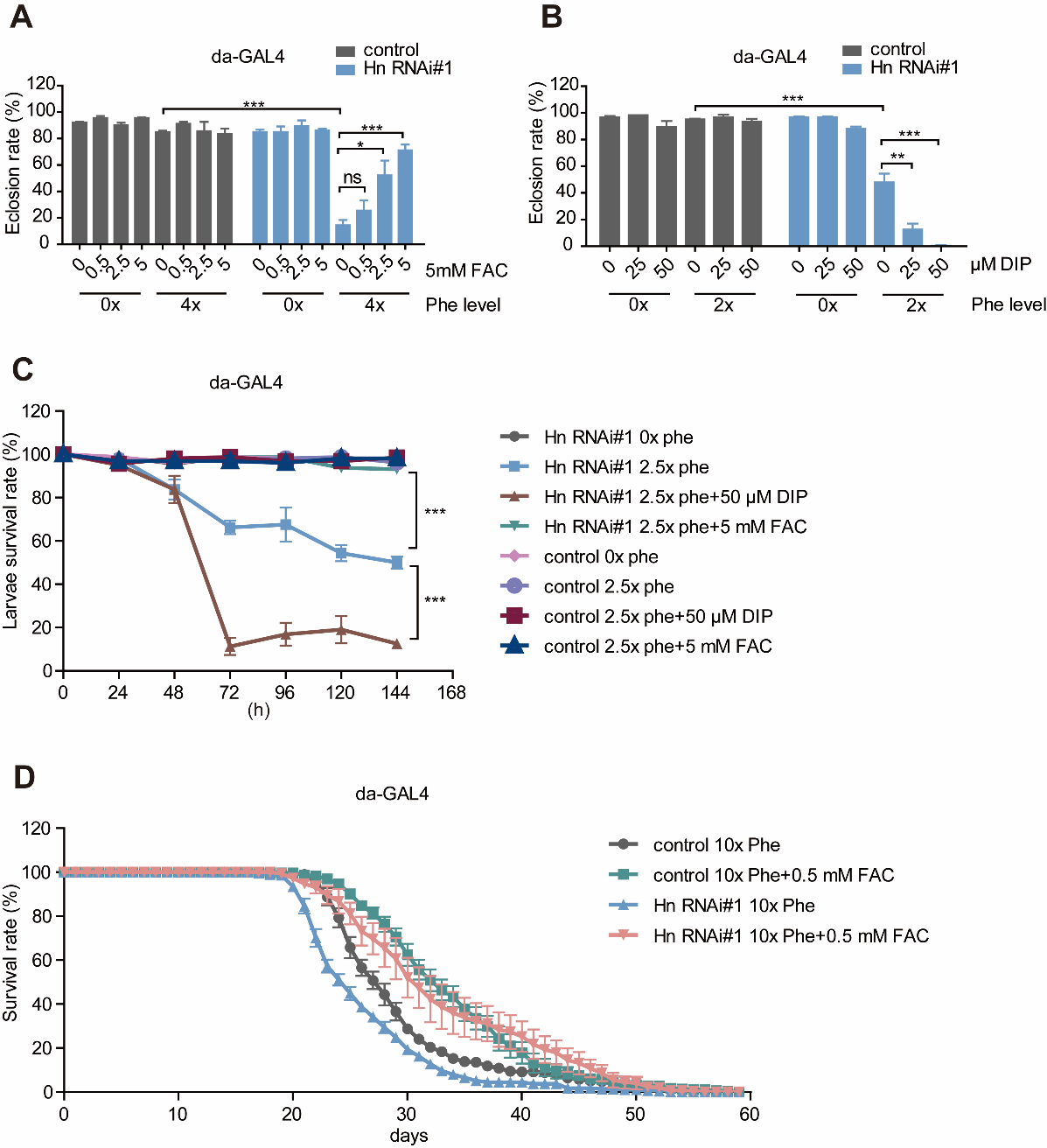


**Fig.S4 Phe-sensitivity of PKU *Drosophila* is rectified by iron supplementation and exacerbated by iron deficiency.** **(A)** The salvage efficacy on the eclosion defect of *Hn* knockdown increased with incremental FAC levels. n = 60 larvae per vial, n = 3 vials per experimental group. **(B)** Eclosion rate of *da-GAL4* > *Hn RNAi#1* dropped with increment of DIP. n = 60 larvae per vial, n ≥ 3 vials per experimental group. **(C)** The larval lethality of *Hn* knockdown depended on the iron levels in the diet. n = 40 larvae per vial, n ≥ 4 vials per experimental group. **(D)** The lifespan of *Hn RNAi#1* driven by *da-GAL4* in 10x Phe food could be partially rescued by 0.5 mM FAC. The adult flies in 10x Phe food were from normal food (1^st^-instar larvae to pupae stage) and the adult flies in 10x Phe + 0.5 mM FAC food were from 0.5 mM FAC food (1^st^-instar larvae to pupae stage) respectively. n = 60 larvae per vial, n = 5 vials per experimental group.


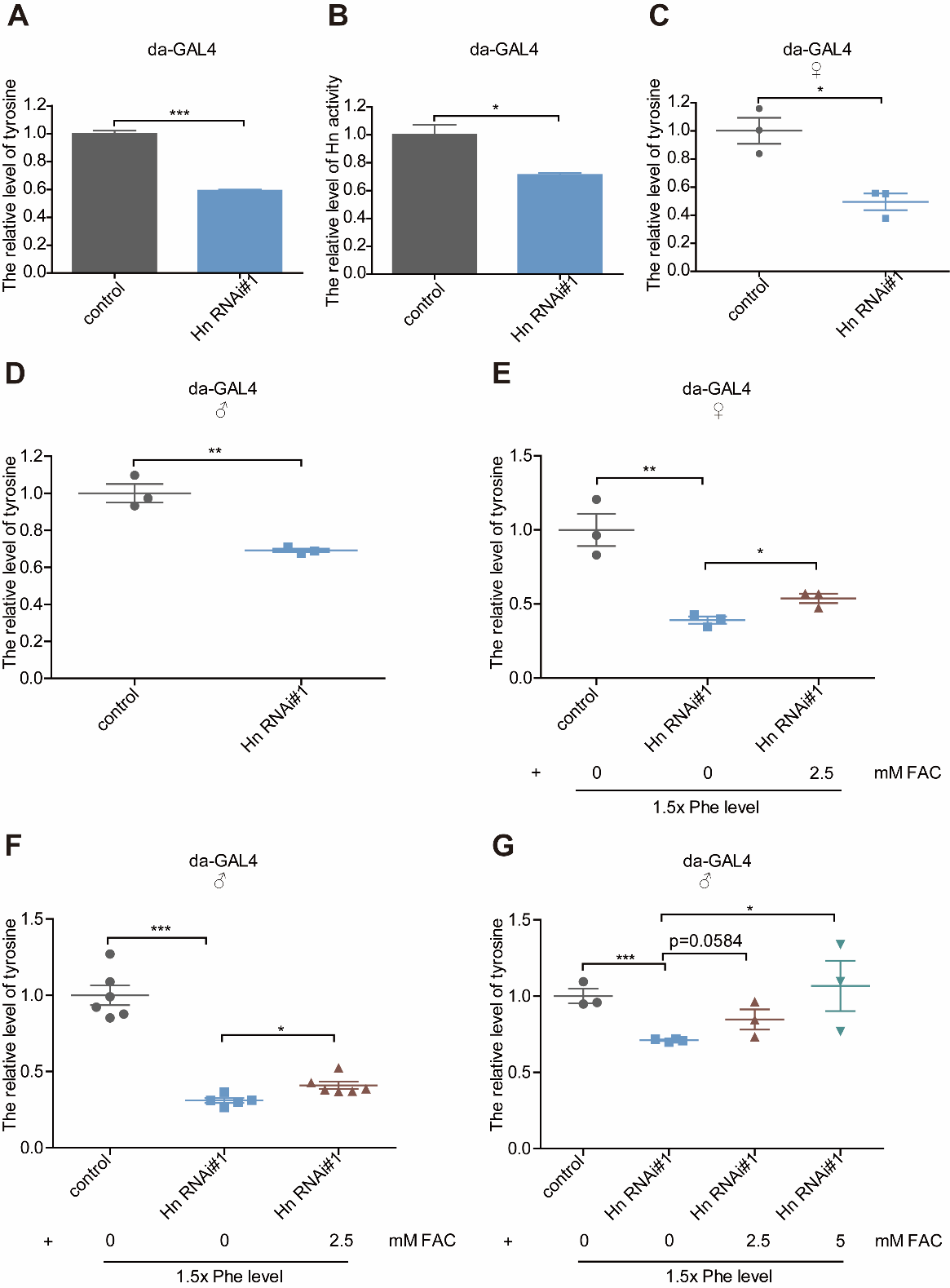


**Fig.S5 Dietary iron impacts bodily tyrosine formation in PKU *Drosophila*.** **(A)** Relative levels of tyrosine decreased after *Hn* knockdown. Genotypes of the 3^rd^-instar larvae used were *da-GAL4* > control (TB072) and *da-GAL4 > Hn RNAi#1* (THU2346). n = 10 3^rd^-instar larvae per parallel experiment, three parallels per experimental group. **(B)** Relative levels of Hn activity decreased after *Hn* knockdown. Genotypes of the 3^rd^-instar larvae used were *da-GAL4* > control (TB072) and *da-GAL4 > Hn RNAi#1* (THU2346). n = 10 3^rd^-instar larvae per parallel experiment, three parallels per experimental group. Relative levels of tyrosine decreased after *Hn* knockdown in whole female **(C)** and male **(D)** adult flies. n = 10 adult flies per parallel experiment, three parallels per experimental group. FAC slightly increased the tyrosine levels in the *Hn* *RNAi* female **(E)** and male **(F)** flies in 1.5x Phe food. n = 10 adult flies per parallel experiment, at least three parallels per experimental group. **(G)** FAC increased the tyrosine level in the *Hn* *RNAi* flies’ male heads in Phe food. n = 100 male flies’ heads per parallel experiment, at least three parallels per experimental group.

**
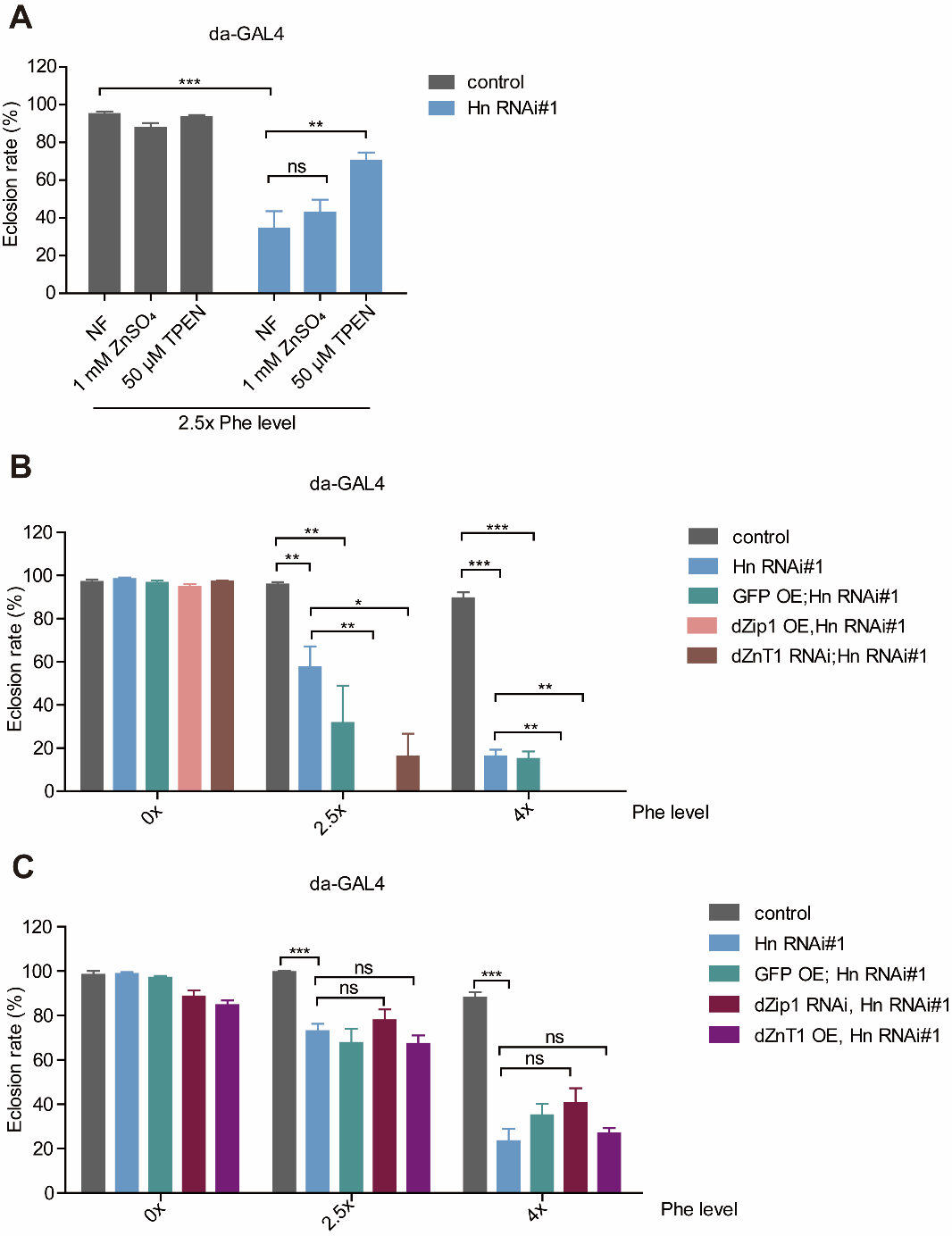
**

**Fig.S6 The Phe-sensitivity of PKU *Drosophila* is moderately affected by genetic modulation of some zinc transporters.** **(A)** TPEN could partially rescue the eclosion rate of *da-GAL4* > *Hn RNAi* on Phe food. n = 60 larvae per vial, n ≥ 3 vials per experimental group. **(B)** The eclosion defect of *Hn* *RNAi* *Drosophila* could be aggravated by *dZip1* OE or *dZnT1* RNAi. Genotypes of the *Drosophila* used were *da-GAL4* > control, *da-GAL4* > *Hn RNAi#1*, *da-GAL4* > *GFP OE; Hn RNAi#1, da-GAL4* > *dZip1 OE, Hn RNAi#1, da-GAL4* > *dZnT1 RNAi; Hn RNAi#1.* n = 60 larvae per vial, n ≥ 3 vials per experimental group. **(C)** *dZip1 RNAi* and *dZnT1 OE* could not rescue the eclosion rate of *Hn* knockdown in whole body. Genotypes of the *Drosophila* used were *da-GAL4* > control, *da-GAL4* > *Hn RNAi#1*, *da-GAL4* > *GFP OE; Hn RNAi#1*, *da-GAL4* > *dZip1 RNAi, Hn RNAi#1*, *da-GAL4* > *dZnT1 OE, Hn RNAi#1*. n = 60 larvae per vial, n ≥ 4 vials per experimental group.


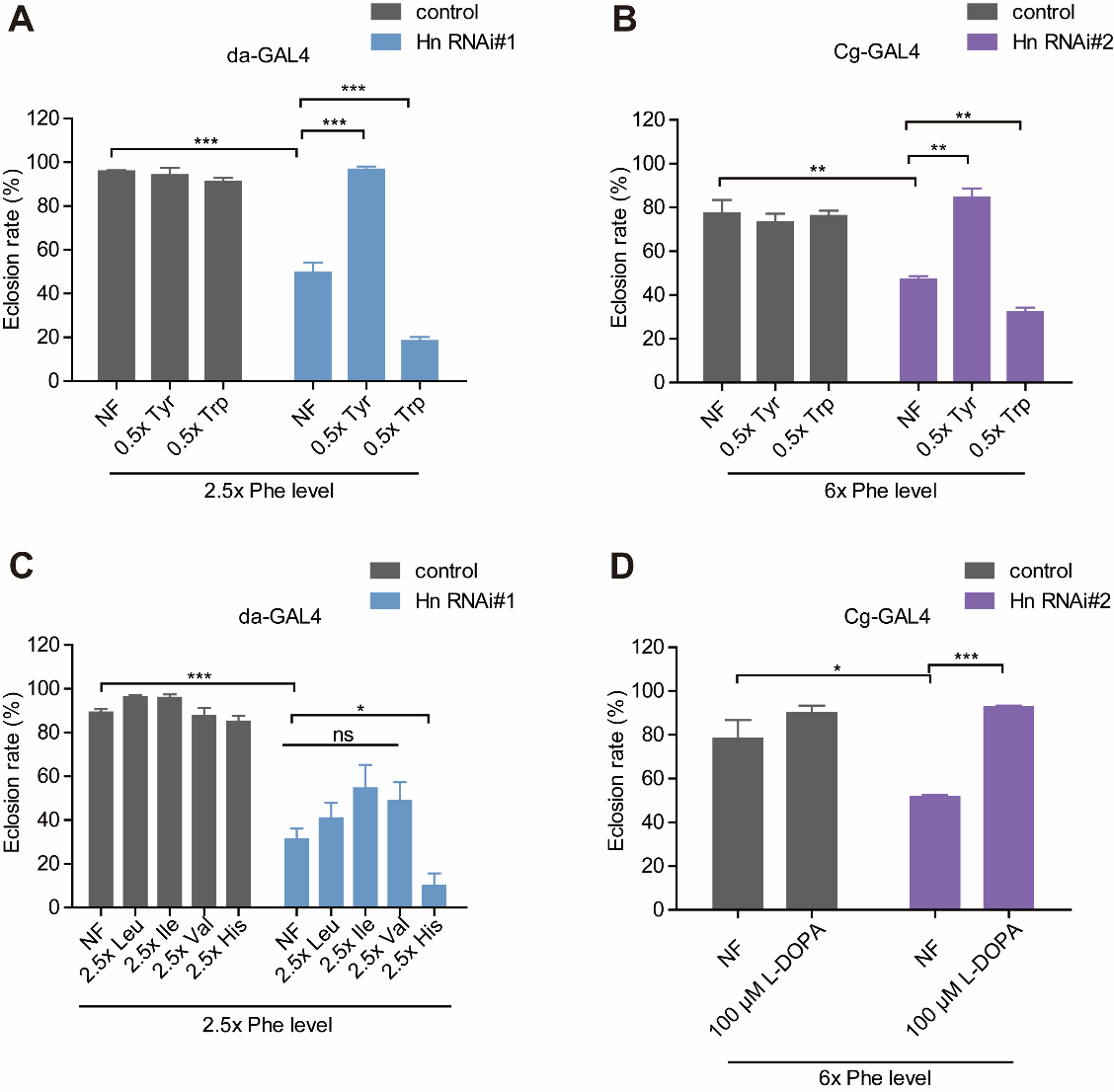


**Fig.S7 Tyrosine and L-DOPA dramatically improve the Phe-sensitivity of PKU *Drosophila*. (A)** 0.5x Tyr supplement rescued the eclosion defect of *da-GAL4* > *Hn RNAi#1* in 2.5x Phe food but 0.5x Trp exacerbated it. n = 60 larvae per vial, n = 4 vials per experimental group. **(B)** 0.5x Tyr and 0.5x Trp respectively increased and decreased the eclosion rate of *Cg-GAL4* > *Hn RNAi* reared in 6x Phe food*.* #2 line was used. n = 60 larvae per vial, n = 3 vials per experimental group. **(C)** 2.5x Leu, 2.5x Ile, 2.5x Val and 2.5x His did not affect the eclosion defect of *da-GAL4* > *Hn RNAi* reared in 2.5x Phe food. #1 line was used. n = 60 larvae per vial, n = 4 vials per experimental group. **(D)** 100 μM L-DOPA rescued the survival of fat body *Hn* knockdown in 6x Phe food. #2 line was used. n = 60 larvae per vial, n = 3 vials per experimental group.

**
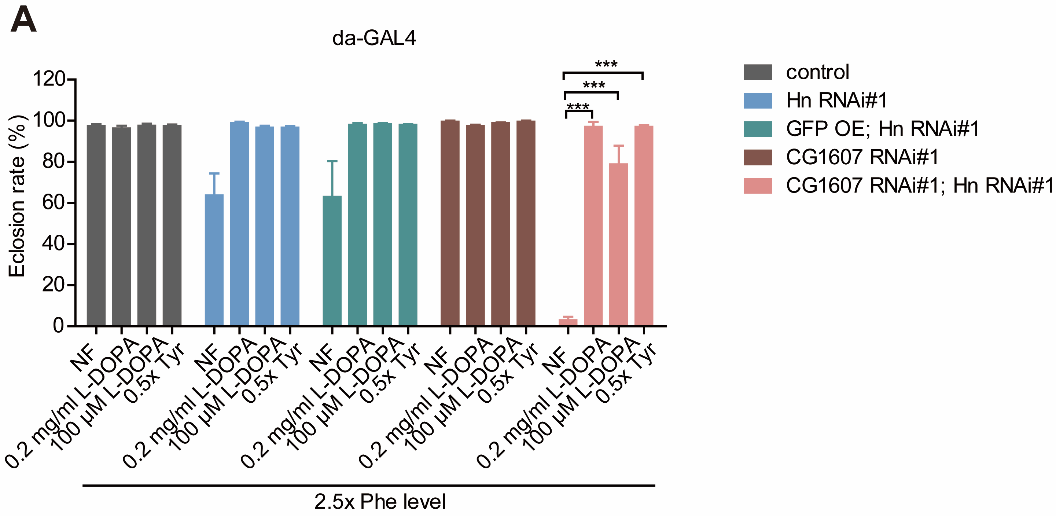
**

**Fig.S8 The eclosion defect of *da-GAL4* > *CG1607 RNAi; Hn RNAi* can be rescued by Tyr and L-DOPA (A)** Tyr and L-DOPA rescued the eclosion defect of *da-GAL4* > *CG1607 RNAi; Hn RNAi.* n = 60 larvae per vial, n ≥ 4 vials per experimental group.


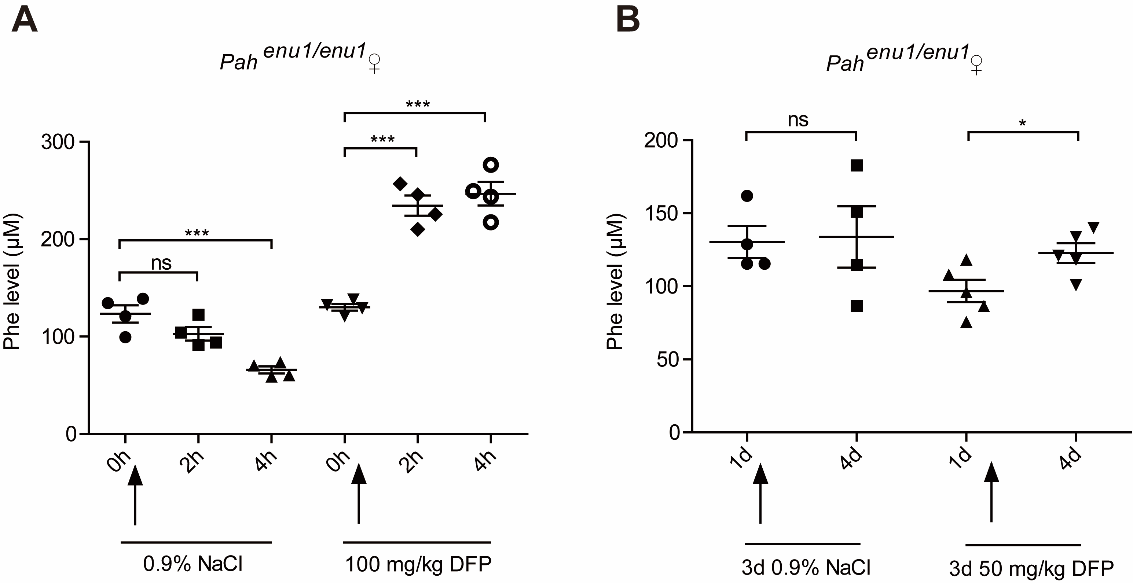


**Fig.S9 Iron chelator DFP increases the Phe levels of *Pah^enu1/enu1^*. (A)** Phenylalanine levels of *Pah^enu1/enu1^* mice after once 100 mg/kg DFP by intraperitoneal injection. 0.9% NaCl was used as the control and the whole period is fasting. n = 4. **(B)** Phenylalanine levels of *Pah^enu1/enu1^* mice after three times 50 mg/kg DFP intraperitoneal injection. n = 4~5.


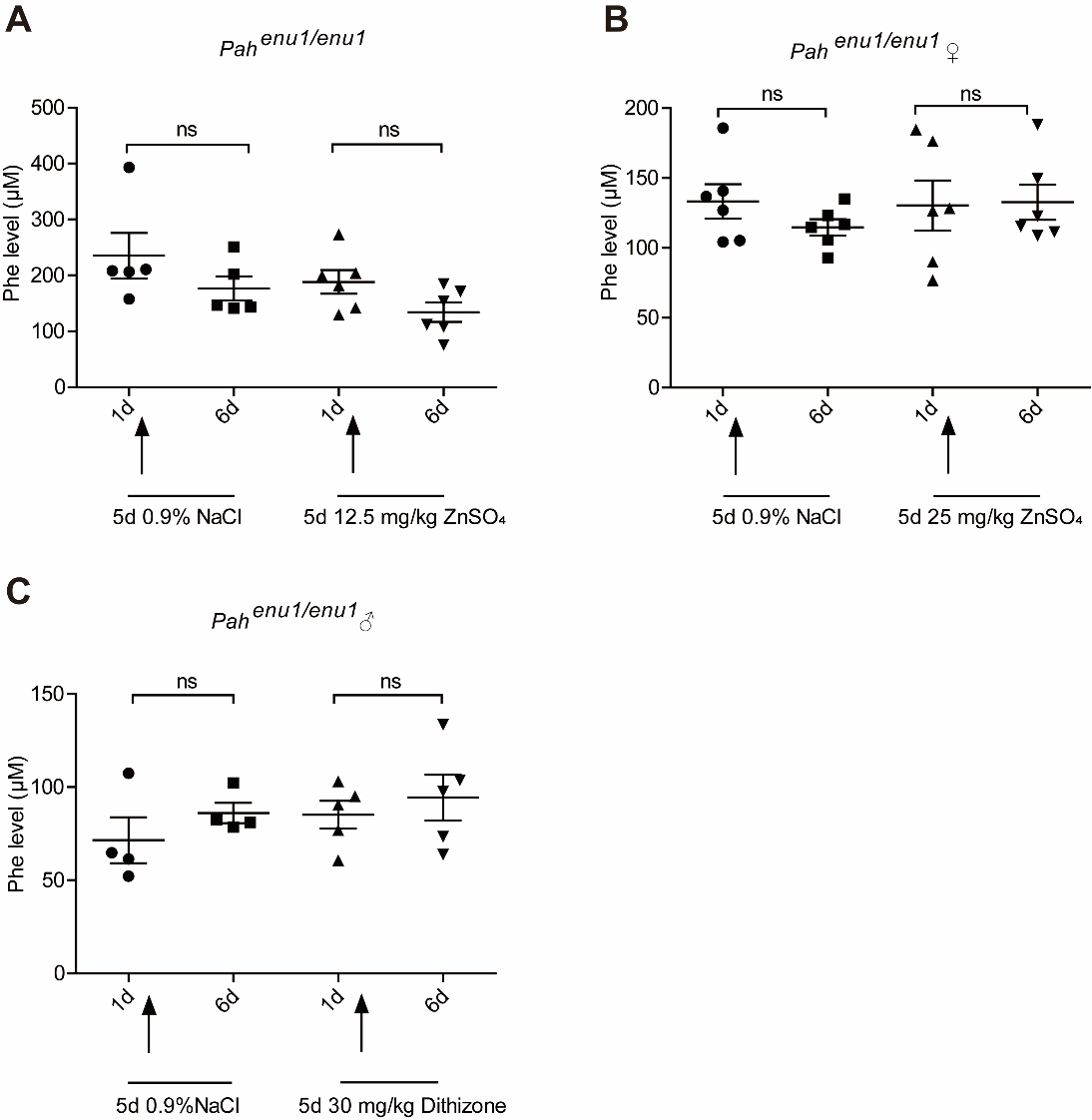


**Fig.S10 Zinc does not obviously impact the blood Phe level in *Pah^enu1/enu1^* mice. (A)** Phenylalanine levels of *Pah^enu1/enu1^* mice after saline and ZnSO_4_ treatment. Five times of intraperitoneal injection were performed to these mice. n = 5~6. **(B)** Phenylalanine levels of *Pah^enu1/enu1^* mice after five times of intragastric administration of saline or zinc. n = 6. **(C)** Phenylalanine levels of *Pah^enu1/enu1^* mice after saline and dithizone treatment for five times of intraperitoneal injection. n = 4~5. For A, B and C, mice were fed on the Rodent Diet, which contains 25 ppm Fe.
