# Supplementary material for: Drosophila phenylketonuria modeling helps reveal the disease etiology and the modulation role of iron
Source: Genes Dis. 2025 Aug 7;13(2):101790. doi: 10.1016/j.gendis.2025.101790 (PMC12664607; doi:10.1016/j.gendis.2025.101790)
Supplement: Multimedia component 2 [file mmc2.pdf]

Supplementary Figures

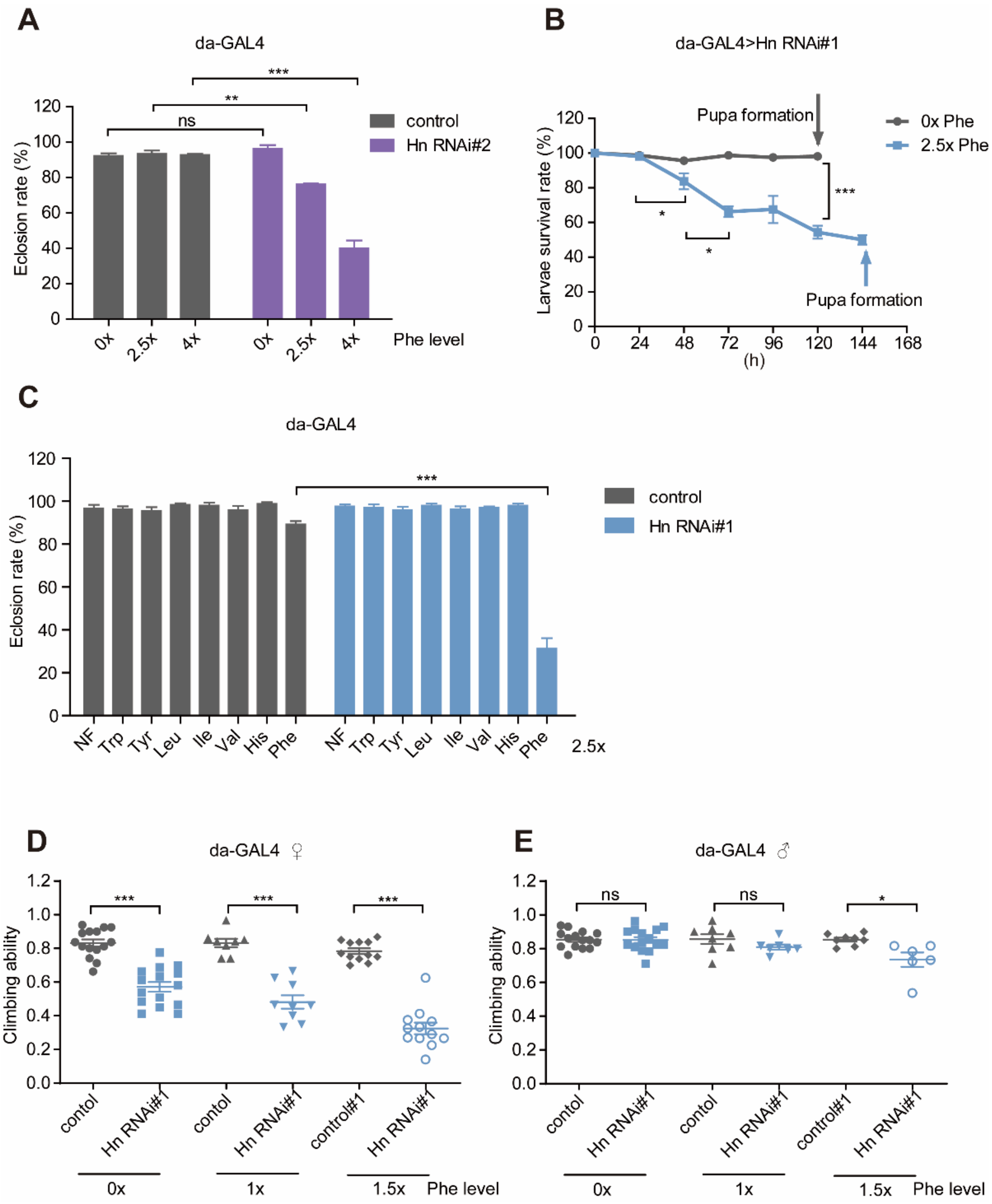

Fig.S1 Ubiquitous *Hn* knockdown confers *Drosophila* phenylalanine sensitivity. (A) Eclosion rates of *da-GAL4* > control (V#w^{1118}) and *da-GAL4* > *Hn RNAi#2* (V#35240) in 0x, 2.5x and 4x Phe foods. n = 60 larvae per vial, n ≥ 3 vials per experimental group. **(B)** Larval lifespan of *Hn* knockdown. 0 h demotes the time when the eggs hatched to the 1st-instar larvae. n = 40 larvae per vial, n ≥ 4 vials per experimental group. **(C)** Eclosion rate of *da-GAL4* > control and *da-GAL4* > *Hn RNAi#1* in food with 2.5x different amino acids including Trp, Tyr, Leu, Ile, Val, His and Phe. n = 60 larvae per vial, n = 3 vials per experimental group. **(D)** *Hn*-konckdown flies displayed climbing defects in 0x, 1x and 1.5x Phe food. n=20 female flies per vial, n≥8 parallels per experimental group. **(E)** *Hn*-konckdown male flies show slightly climbing defects in 1.5x Phe food. n=20 male flies per vial, n≥6 parallels per experimental group.

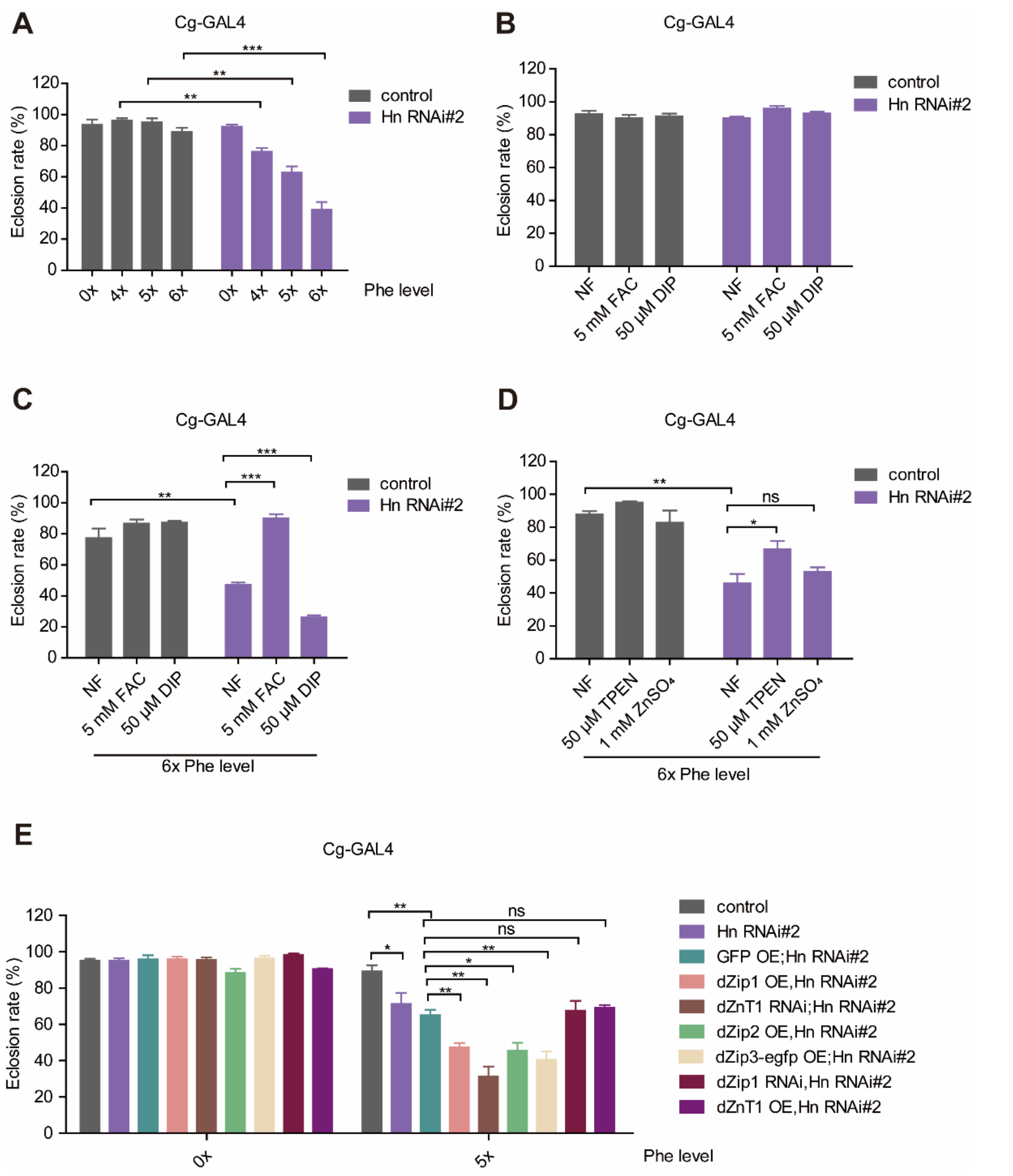

Fig.S2 *Hn* knockdown in *Drosophila* fat body reproduces the phenotypes of the whole-body knockdown. (A) Fat-specific Hn knockdown subjected *Drosophila* to Phe toxicity. Eclosion rates of *Cg-GAL4* > control (V#w^{1118}) and *Cg-GAL4* > *Hn RNAi#2* (V#35240) in food with different Phe levels (0x, 4x, 5x, 6x) were analyzed. n = 60 larvae per vial, n = 3 vials per experimental group. **(B)** In normal food, dietary iron did not affect the eclosion of the fat-specific *Hn* knockdown *Drosophila*. *Cg-GAL4* > control and *Cg-GAL4* > *Hn RNAi#2 Drosophila* were tested. DIP was used the iron chelator. n = 60 larvae per vial, n = 4 vials per experimental group. **(C)** FAC supplement rescued, while iron chelation worsened, the eclosion defect of the fat-specific *Hn*-knockdown *Drosophila*. *Cg-GAL4* > control and *Cg-GAL4* > *Hn RNAi#2* in 6x Phe food was tested. n = 60 larvae per vial, n = 3 vials per experimental group. **(D)** Zinc modulation only mildly influenced the fat-specific *Hn*-knockdown *Drosophila*. 50 μM TPEN could slightly rescue the eclosion rate of *Cg-GAL4* > *Hn RNAi#2* in 6x Phe food. n = 60 larvae per vial, n ≥ 3 vials per experimental group. **(E)** The eclosion defect of the fat-specific *Hn RNAi Drosophila* could be moderately aggravated by zinc accumulation

mediated by genetic measures. *Drosophila* was tested on 5x Phe food and the zinc homeostasis was interfered with *dZip1* OE, *dZnT1* RNAi, *dZip2* OE, *dZip3-egfp* OE, *dZip1 RNAi or dZnT1 OE*. Genotypes of the flies used were *Cg-GAL4* > control, *Cg-GAL4 > Hn RNAi#2*, *Cg-GAL4 > GFP OE; Hn RNAi#2*, *Cg-GAL4 > dZip1 OE, Hn RNAi#2*, *Cg-GAL4 > dZnT1 RNAi; Hn RNAi#2*, *Cg-GAL4 > dZip2 OE, Hn RNAi#2*, *Cg-GAL4 > dZip3-egfp OE; Hn RNAi#2*, *Cg-GAL4 > dZip1 RNAi, Hn RNAi#2*, *Cg-GAL4 > dZnT1 OE, Hn RNAi#2.* n = 60 larvae per vial, n = 4 vials per experimental group.

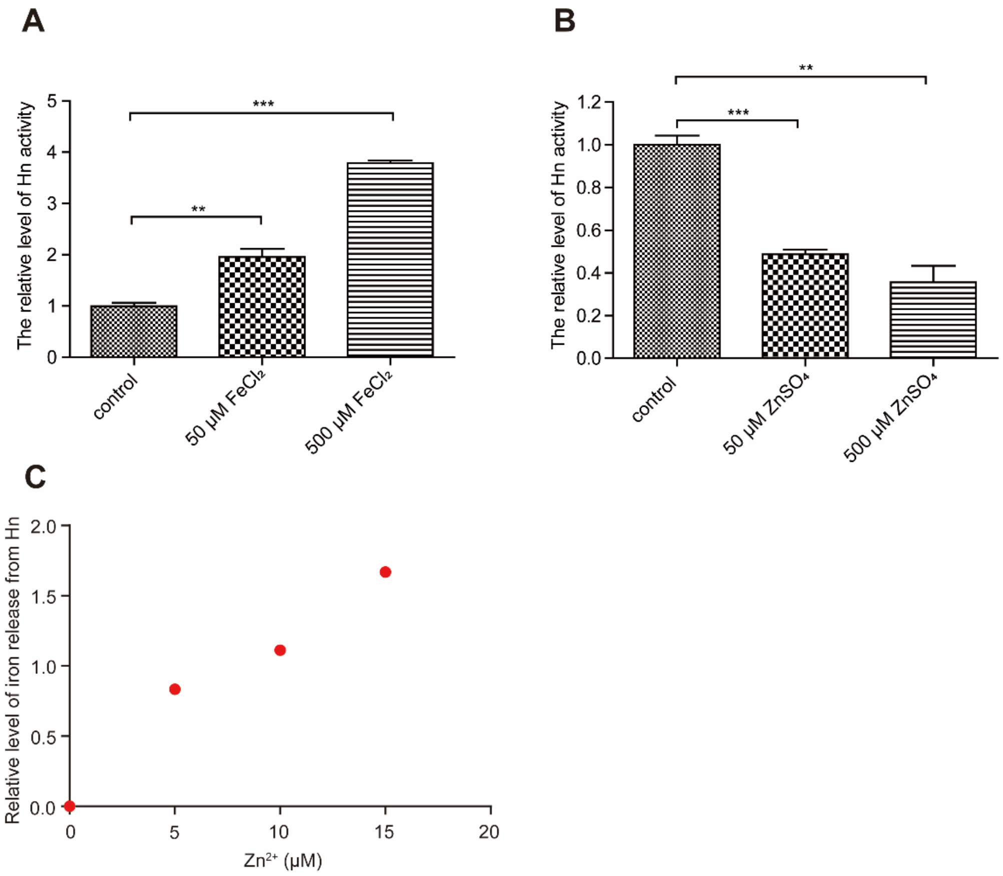

Fig.S3 Hn activity is potently regulated by iron and to a lesser extent by zinc *in vitro*. (**A**) The activity of recombinant Hn from *E. coli* was up-regulated by Fe^{2+}. **(B)** The activity of recombinant Hn from *E. coli* was down-regulated by Zn^{2+}. **(C)** Iron ion release from Hn by zinc ion. Hn was incubated with 15 μM ferrous iron and different concentrations of Zn^{2+}. Free and bound metal ions were separated by ultrafiltration, and the iron level in filtrate was determined by ICP-MS. Free iron increased with increased zinc concentrations in the buffer.

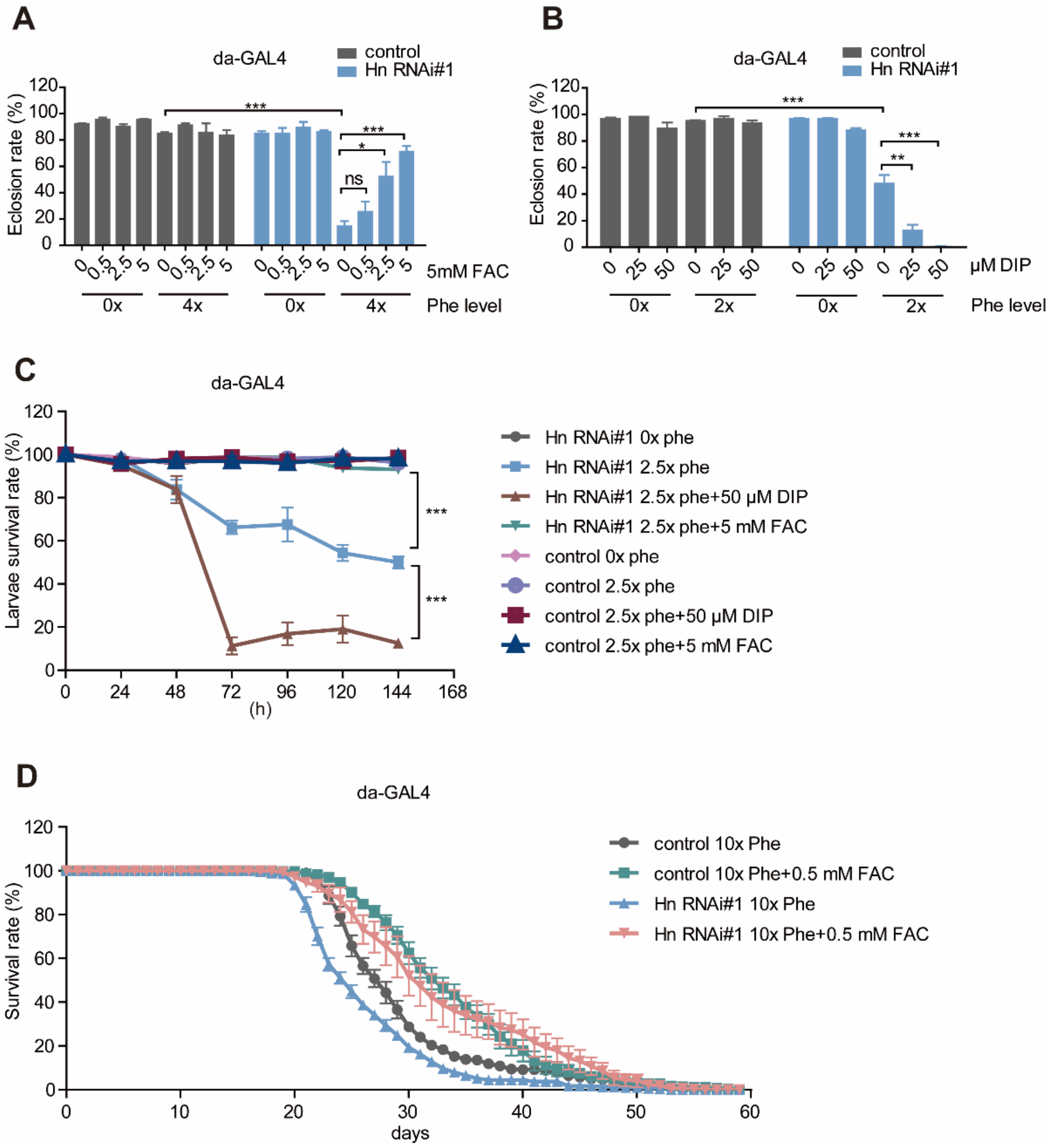

Fig.S4 Phe-sensitivity of PKU *Drosophila* is rectified by iron supplementation and exacerbated by iron deficiency. (A) The salvage efficacy on the eclosion defect of *Hn* knockdown increased with incremental FAC levels. n = 60 larvae per vial, n = 3 vials per experimental group. **(B)** Eclosion rate of *da-GAL4 > Hn RNAi#1* dropped with increment of DIP. n = 60 larvae per vial, n ≥ 3 vials per experimental group. **(C)** The larval lethality of *Hn* knockdown depended on the iron levels in the diet. n = 40 larvae per vial, n ≥ 4 vials per experimental group. **(D)** The lifespan of *Hn RNAi#1* driven by *da-GAL4* in 10x Phe food could be partially rescued by 0.5 mM FAC. The adult flies in 10x Phe food were from normal food (1st-instar larvae to pupae stage) and the adult flies in 10x Phe + 0.5 mM FAC food were from 0.5 mM FAC food (1st-instar larvae to pupae stage) respectively. n = 60 larvae per vial, n = 5 vials per experimental group.

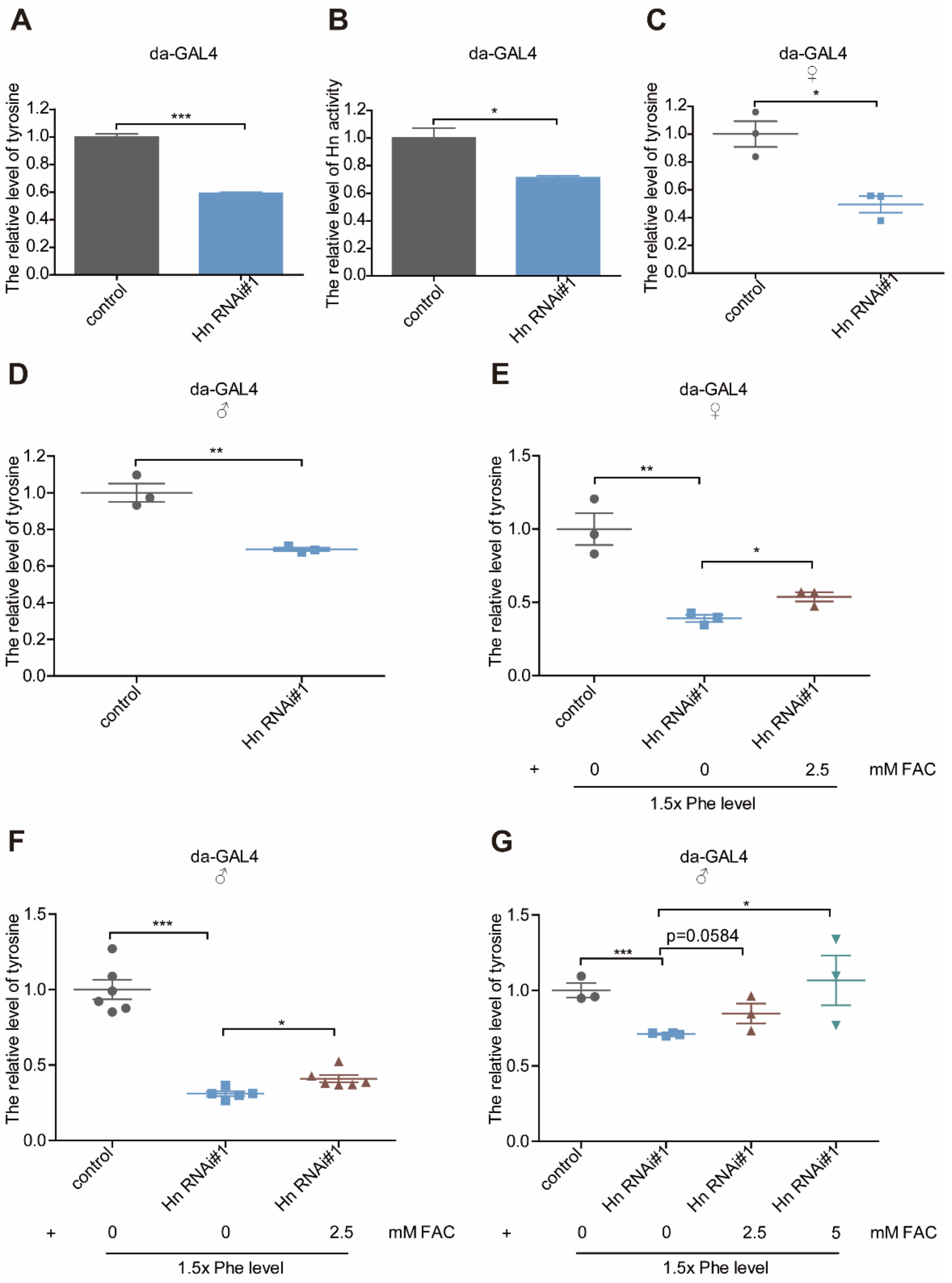

Fig.S5 Dietary iron impacts bodily tyrosine formation in PKU *Drosophila*. (A) Relative levels of tyrosine decreased after *Hn* knockdown. Genotypes of the 3rd-instar larvae used were *da-GAL4* > control (TB072) and *da-GAL4* > *Hn RNAi#1* (THU2346). n = 10 3rd-instar larvae per parallel experiment, three parallels per experimental group. **(B)** Relative levels of Hn activity decreased after *Hn* knockdown. Genotypes of the 3rd-instar larvae used were *da-GAL4* > control (TB072) and *da-GAL4* > *Hn RNAi#1* (THU2346). n = 10 3rd-instar larvae per parallel experiment, three parallels per experimental group. Relative levels of tyrosine decreased after *Hn* knockdown in whole female **(C)** and male **(D)** adult flies. n = 10 adult flies per parallel experiment, three parallels per experimental group. FAC slightly increased the tyrosine levels in the *Hn RNAi* female **(E)** and male **(F)** flies in 1.5x Phe food. n = 10 adult flies per parallel experiment, at least three parallels per

experimental group. **(G)** FAC increased the tyrosine level in the *Hn RNAi* flies' male heads in Phe food. n = 100 male flies' heads per parallel experiment, at least three parallels per experimental group.

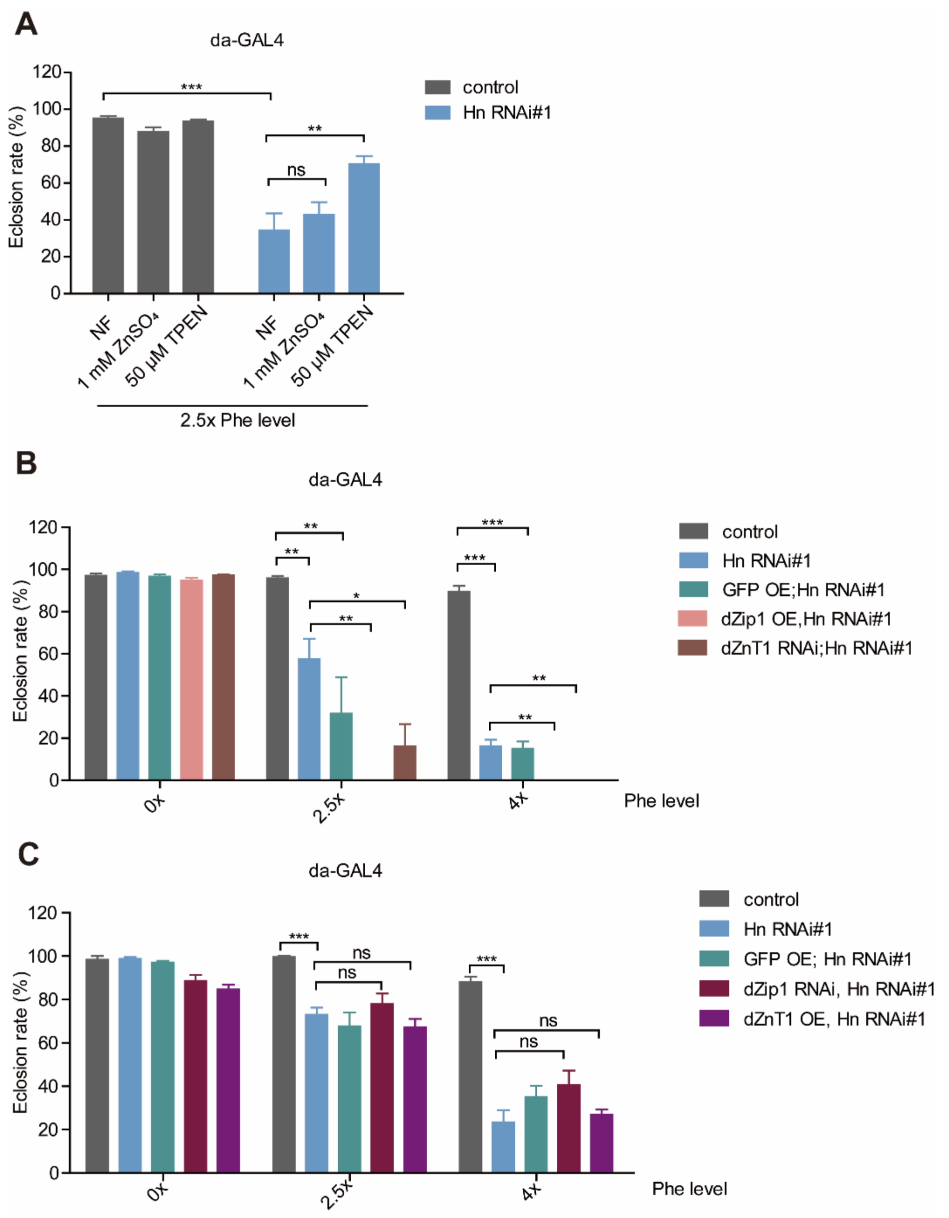

Fig.S6 The Phe-sensitivity of PKU *Drosophila* is moderately affected by genetic modulation of some zinc transporters. (A) TPEN could partially rescue the eclosion rate of *da-GAL4 > Hn RNAi* on Phe food. n = 60 larvae per vial, n ≥ 3 vials per experimental group. **(B)** The eclosion defect of *Hn RNAi Drosophila* could be aggravated by *dZip1* OE or *dZnT1* RNAi. Genotypes of the *Drosophila* used were *da-GAL4 >* control, *da-GAL4 > Hn RNAi#1*, *da-GAL4 > GFP OE; Hn RNAi#1, da-GAL4 > dZip1 OE, Hn RNAi#1, da-GAL4 > dZnT1 RNAi; Hn RNAi#1.* n = 60 larvae per vial, n ≥ 3 vials per experimental group. **(C)** *dZip1 RNAi* and *dZnT1 OE* could not rescue the eclosion rate of *Hn* knockdown in whole body. Genotypes of the *Drosophila* used were *da-GAL4 >* control, *da-GAL4 > Hn RNAi#1*, *da-GAL4 > GFP OE; Hn RNAi#1*, *da-GAL4 > dZip1 RNAi, Hn RNAi#1*, *da-GAL4 > dZnT1 OE, Hn RNAi#1*. n = 60 larvae per vial, n ≥ 4 vials per experimental group.

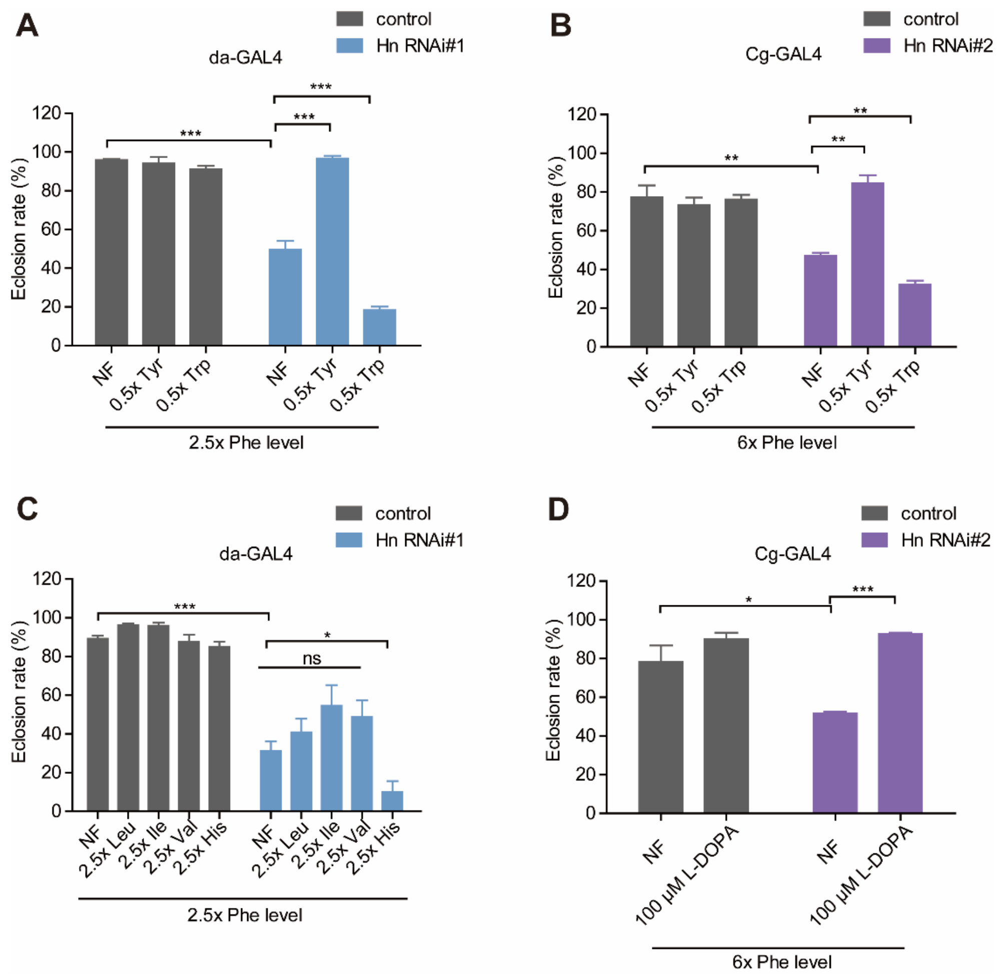

Fig.S7 Tyrosine and L-DOPA dramatically improve the Phe-sensitivity of PKU *Drosophila*. (A) 0.5x Tyr supplement rescued the eclosion defect of *da-GAL4 > Hn RNAi#1* in 2.5x Phe food but 0.5x Trp exacerbated it. n = 60 larvae per vial, n = 4 vials per experimental group. **(B)** 0.5x Tyr and 0.5x Trp respectively increased and decreased the eclosion rate of *Cg-GAL4 > Hn RNAi* reared in 6x Phe food. #2 line was used. n = 60 larvae per vial, n = 3 vials per experimental group. **(C)** 2.5x Leu, 2.5x Ile, 2.5x Val and 2.5x His did not affect the eclosion defect of *da-GAL4 > Hn RNAi* reared in 2.5x Phe food. #1 line was used. n = 60 larvae per vial, n = 4 vials per experimental group. **(D)** 100 μM L-DOPA rescued the survival of fat body *Hn* knockdown in 6x Phe food. #2 line was used. n = 60 larvae per vial, n = 3 vials per experimental group.

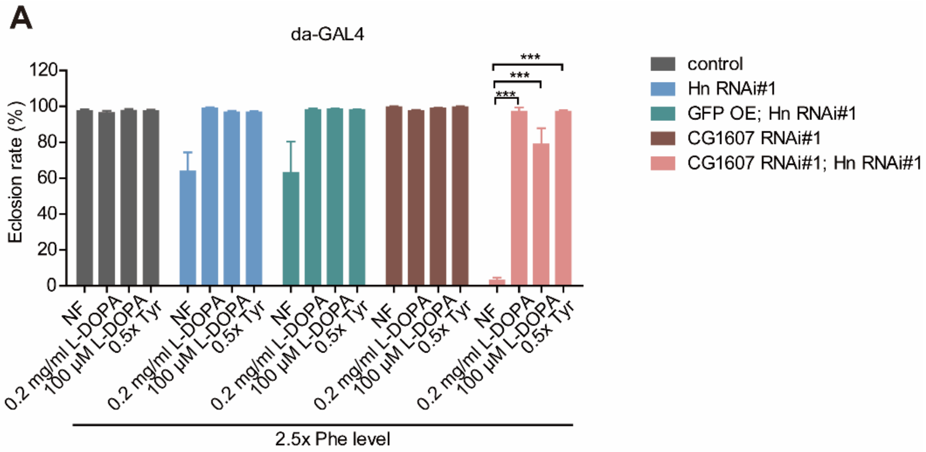

Fig.S8 The eclosion defect of *da-GAL4 > CG1607 RNAi; Hn RNAi* can be rescued by Tyr and L-DOPA (A) Tyr and L-DOPA rescued the eclosion defect of *da-GAL4 > CG1607 RNAi; Hn RNAi.* n = 60 larvae per vial, n ≥ 4 vials per experimental group.

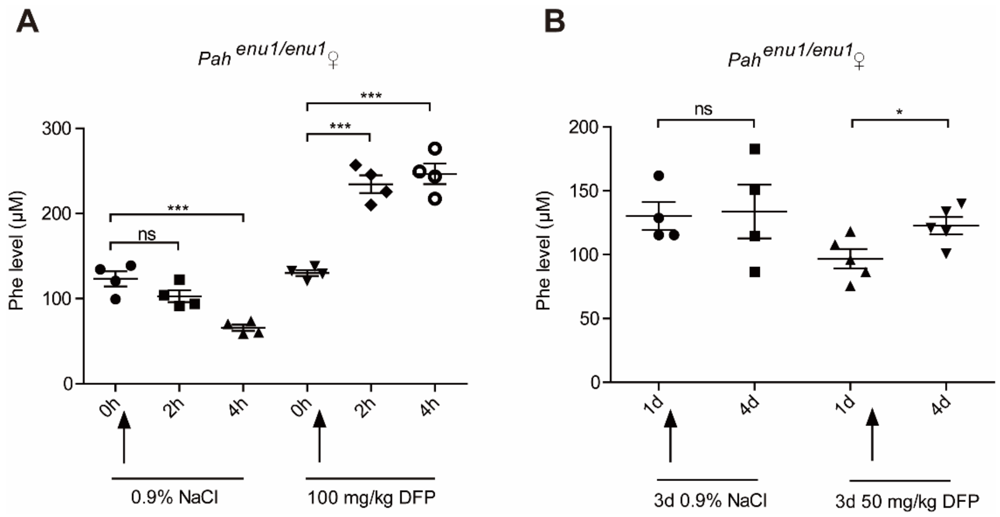

Fig.S9 Iron chelator DFP increases the Phe levels of *Pah*$^{enu1/enu1}$. (A) Phenylalanine levels of *Pah*$^{enu1/enu1}$ mice after once 100 mg/kg DFP by intraperitoneal injection. 0.9% NaCl was used as the control and the whole period is fasting. n = 4. **(B)** Phenylalanine levels of *Pah*$^{enu1/enu1}$ mice after three times 50 mg/kg DFP intraperitoneal injection. n = 4~5.

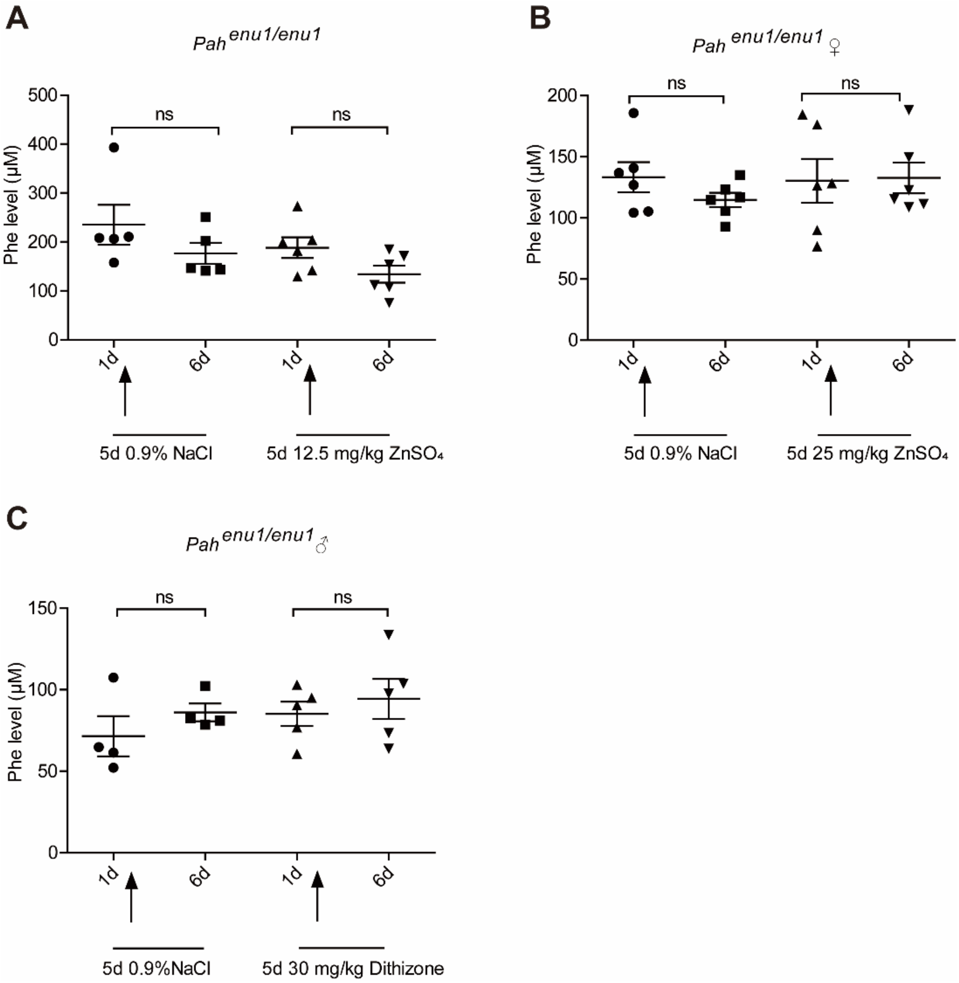

Fig.S10 Zinc does not obviously impact the blood Phe level in *Pah*$^{enu1/enu1}$ mice. (A) Phenylalanine levels of *Pah*$^{enu1/enu1}$ mice after saline and $ZnSO_4$ treatment. Five times of intraperitoneal injection were performed to these mice. n = 5~6. **(B)** Phenylalanine levels of *Pah*$^{enu1/enu1}$ mice after five times of intragastric administration of saline or zinc. n = 6. **(C)** Phenylalanine levels of *Pah*$^{enu1/enu1}$ mice after saline and dithizone treatment for five times of intraperitoneal injection. n = 4~5. For A, B and C, mice were fed on the Rodent Diet, which contains 25 ppm Fe.
